# AP‐1 is a regulatory transcription factor of inflammaging in the murine kidney and liver

**DOI:** 10.1111/acel.13858

**Published:** 2023-05-08

**Authors:** Xiaojie Yu, Yuting Wang, Yifan Song, Xianda Gao, Hongkui Deng

**Affiliations:** ^1^ The MOE Key Laboratory of Cell Proliferation and Differentiation, College of Life Sciences, Peking‐Tsinghua Center for Life Sciences Peking University Beijing China; ^2^ School of Basic Medical Sciences, State Key Laboratory of Natural and Biomimetic Drugs Peking University Beijing China

**Keywords:** aging, AP‐1, inflammation, kidney, liver, transcription factor

## Abstract

Aging is characterized by chronic low‐grade inflammation in multiple tissues, also termed “inflammaging”, which represents a significant risk factor for many aging‐related chronic diseases. However, the mechanisms and regulatory networks underlying inflammaging across different tissues have not yet been fully elucidated. Here, we profiled the transcriptomes and epigenomes of the kidney and liver from young and aged mice and found that activation of the inflammatory response is a conserved signature in both tissues. Moreover, we revealed links between transcriptome changes and chromatin dynamics through integrative analysis and identified AP‐1 and ETS family transcription factors (TFs) as potential regulators of inflammaging. Further in situ validation showed that c‐JUN (a member of the AP‐1 family) was mainly activated in aged renal and hepatic cells, while increased SPI1 (a member of the ETS family) was mostly induced by elevated infiltration of macrophages, indicating that these TFs have different mechanisms in inflammaging. Functional data demonstrated that genetic knockdown of *Fos*, a major member of the AP‐1 family, significantly attenuated the inflammatory response in aged kidneys and livers. Taken together, our results revealed conserved signatures and regulatory TFs of inflammaging in the kidney and liver, providing novel targets for the development of anti‐aging interventions.

## Graphical abstract

In this study, AP‐1 and ETS family transcription factors (TFs) are identified as potential regulators of inflammaging, while HNFs and PPARs act as negative regulators contributing to dysregulated metabolism in the kidney and liver during aging. Additionally, AP‐1 and ETS have different roles in regulating inflammation, and genetic manipulation of AP‐1 significantly attenuates the inflammatory response in aged kidneys and livers.

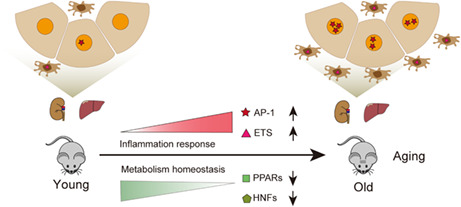

**Abbreviations**
AAV: adeno‐associated virus
AP‐1: activator protein 1
ATAC‐seq: assay for transposase‐accessible chromatin using sequencing
CD: chromatin dependence
COI: chromatin opening index
ETS: E26 transformation‐specific
FDR: false‐discovery rate
GO: gene ontology
GSEA: gene set enrichment analysis
IF: immunofluorescence
IGV: integrative genomics viewer
IHC: immunohistochemistry
PCA: principal component analysis
SA‐β‐gal: senescence‐associated β‐galactosidase
TFBS: transcription factor binding site
TF: transcription factor
TSS: transcription start site

## 1 INTRODUCTION

Aging is characterized by chronic low‐grade inflammation, which is also referred to as “inflammaging” (Lopez‐Otin et al., 2013; Xia et al., 2016). Increasing evidence indicates that inflammaging is closely linked to the progression of many aging‐related diseases, such as atherosclerosis, diabetes, neurodegenerative disorders, and cancer (Ferrucci & Fabbri, 2018; Franceschi & Campisi, 2014; Fulop et al., 2018). Deepening the understanding of the regulatory mechanisms underlying inflammaging is important to facilitate the modulation of its negative effects and lengthen health span.

A prominent feature of inflammaging is its systematic nature (Xia et al., 2016). Increased pro‐inflammatory cytokines produced by tissues during aging affect the normal physiological function of the tissue, as well as other tissues, finally leading to a loss of homeostasis (Mancuso & Bouchard, 2019). The kidney and liver play a central role in the maintenance of whole‐body homeostasis by regulating energy metabolism, waste excretion, and biosynthesis (Moestrup & Nielsen, 2005; Rui, 2014). Aging‐associated liver degeneration is linked to increased levels of proinflammatory cytokines and a higher number of infiltrated immune cells (Jin et al., 2020). Similarly, kidney aging is accompanied by increased inflammatory factors and fibrosis, which results in morphological changes and tissue dysfunction (Kanasaki et al., 2012). Nevertheless, the mechanisms and regulatory networks underlying inflammaging of the kidney and liver remain poorly understood.

Due to the inherent complexity of inflammaging, gaining a mechanistic understanding necessitates an integrative analysis of the transcriptome and epigenome across different tissues. Global alternations in gene expression are a prominent signature of aging and have been widely used to demonstrate the intervention effects of aging delay or acceleration (Browder et al., 2022; Landsberger et al., 2022; Lee et al., 1999). Although most studies describe transcriptional signatures, epigenetic alterations are key bridges between genomic information and transcriptional regulation (Gibney & Nolan, 2010; Zhang et al., 2020). Many aging‐associated changes occur in the chromatin architecture, yet their relevance to gene signatures remains to be established (Sen et al., 2016; Wang, Liu, Hu, et al., 2022). Several studies have explored chromatin dynamics and gene expression changes during mammalian aging (e.g., in muscle stem cells and CD8^+^ T cells), providing important insights into epigenetic remodeling with age (Dong et al., 2022; Ucar et al., 2017). However, most age‐related studies have focused on only one tissue and pairwise comparisons are scarce. It is therefore not clear whether aging in different tissues shares a common mechanism.

In this study, we generated transcriptome (via RNA‐seq) and epigenome (via ATAC‐seq) profiles to explore the common signatures and regulatory factors underlying aging in the kidney and liver. We revealed that the enhanced inflammatory response was the mainly common signature across tissues, and the upregulation of inflammation‐related genes was accompanied by increased chromatin accessibility at promoters. Moreover, transcription factor and regulatory network analyses indicated that AP‐1 and ETS were the key regulators for enhanced inflammation in aging. We validated that activation of AP‐1 was mainly distributed in aged renal and hepatic cells, not in immune cells, and knockdown of AP‐1 attenuated inflammatory response in the aged kidney and liver. Taken together, these results provide insights into the novel regulatory targets of inflammaging, as well as potential therapeutic targets for the alleviation of aging‐related chronic diseases.

## 2 METHODS

### 2.1 Animal experiments

All animal procedures were performed according to NIH guidelines. All practices related to mice were approved by the Institutional Animal Care and Use Committee of Peking University. Female C57BL/6 mice were purchased from Charles River and housed under standard conditions in a specific pathogen‐free, temperature‐controlled environment with a 12‐h day/night cycle until they reached the appropriate age for the experiments.

For animal tissue collection, the kidney and liver samples were obtained from young (2–3 months old) and aged (23–25 months old) female C57BL/6 mice, quickly frozen in liquid nitrogen, and stored at −80°C for RNA or protein extraction and ATAC‐seq library preparation.

For AAV‐infection experiments, 24‐month‐old female C57BL/6 mice were randomized to receive AAV8 sh*Fos* or non‐targeting (NT) vector by intravenous injection. After 3 months, the kidney and liver were collected for analysis.

### 2.2 RNA isolation and RNA‐seq library preparation

TRIzol™ reagent (Sigma‐Aldich, T9424) was used to extract total RNA according to the manufacturer's instructions. Subsequently, the RNA samples were sent to Novogene for cDNA library construction and Illumina sequencing with paired‐end reads on a HiSeq PE150 system.

### 2.3 Nuclei isolation and ATAC‐seq library preparation

Nuclei were isolated from the kidney and liver, and ATAC‐seq libraries were prepared according to the previously described protocol (Corces et al., 2017). Briefly, frozen tissue samples were thawed and homogenized in cold homogenization buffer on ice. Next, 400 μL of the nuclei suspension was subjected to density gradient centrifugation. After centrifugation, the nuclei band was collected, and 50,000 nuclei were transferred into a fresh tube after counting. The nuclei were pelleted at 500 RCF for 10 min at 4°C in a fixed angle centrifuge. After carefully removing the supernatant, transposition was performed by resuspending the nuclei pellet in 50 μL of Transposition Mix containing 1× TD Buffer (20 mM Tris–HCl pH at 7.6, 10 M MgCl_2_ and 20% dimethyl formamide) and 2.5 μL Tn5 (Vazyme, TD501) for 30 min at 37°C. Next, DNA was extracted with 2 × DNA binding beads (Vazyme, N411‐01). Libraries were produced by PCR, for which the cycle numbers were determined by QPCR according to a previous publication (Buenrostro et al., 2015). The final PCR reaction was purified using 0.5–1.2 × DNA binding beads. Library quality was assessed with an Agilent Bioanalyzer 2100, and paired end sequencing was performed with an Illumina Hiseq PE150. Typically, 40–70 million high‐quality reads per library were required.

### 2.4 RNA‐seq data analysis

Sequencing adapters and low‐quality reads were removed from the raw RNA‐seq reads with Trimmomatic (V0.39), with the main parameters set as follows: LEADING:3 TRAILING:3 SLIDINGWINDOW:4:15 MINLEN:26. The trimmed reads were then mapped to the mm10 mouse genome using Hisat2 (V2.2.1). Next, we used HTSeq‐count (V0.12.4) to calculate the count of reads for each gene, with the main parameters set as: ‐f bam ‐s no ‐r pos. The reference genome was downloaded from the USCS Table Browser. To quantitate gene expression, the read count for each gene was normalized using DESeq2 (V1.22.2). Aging‐associated genes were filtered with the following criteria: adjusted *p* value <0.05 and fold change >2 or <0.5.

### 2.5 ATAC‐seq data analysis

The ATAC‐seq data were pre‐processed (trimmed, aligned, filtered, and quality controlled) using the ATAC‐seq pipeline from the Kundaje lab (https://github.com/ENCODE‐DCC/atac‐seq‐pipeline). In the pipeline, the input JSON file included genomic data files and parameters for running the pipeline. Using the auto‐detected adapter mode to trim the reads, we set the parameters to “‐e 0.1 ‐m 5.” Bowtie2 was used to align the reads to the reference genome. The parameters for mapping were set as “‐X2000 ‐‐mm –local.” After the alignment, duplicates were removed by Picard and only uniquely mapped alignments (MAPQ > 10) were kept. MACS2 was used for peak calling, and the parameters for MACS2 were set as “‐g mm ‐B ‐‐shift ‐100 ‐‐extsize 200 –nomodel.” The “norrowpeak” files were used for downstream analysis. The peaks that appeared in more than 60% of the replicate samples in each group were kept for feature counts and subsequent analysis using DiffBind (V2.12.0). The read counts for each peak were normalized using DESeq2 (V1.22.2). Peaks that differed significantly between young and aged samples were identified using DESeq2 with the following criteria: adjusted *p* value <0.05. A gene was identified as a peak‐related gene when the distance from its TSS to the peak was less than 1 kb.

### 2.6 Motif enrichment analysis

Motif enrichment analysis was performed based on selected genome regions using HOMER. For the ATAC‐seq peak‐based motif analysis, the parameters were set to “‐len 8,10,12,15 ‐p 8 ‐S 40.”

### 2.7 TF footprint analysis based on ATAC‐seq data

In the ATAC‐seq method, the Tn5 enzyme recognizes and cleaves DNA in open regions, which causes enhanced alignment of reads in these regions. However, the presence of TFs bound to DNA prevents Tn5 from cleaving DNA at TF binding sites, leaving small regions, referred to as footprints, where the read coverage drops sharply within peak regions with high coverage (Li et al., 2019). For the TF footprint analysis, RGT‐HINT (V0.13.0) footprinting was used with the parameters set as follows: ‐‐atac‐seq ‐‐paired‐end ‐‐organism=mm10.

### 2.8 GSEA analysis

For GSEA analysis, hallmark gene sets (V7.4) and other aging or senescence associated gene sets used in this paper were downloaded from MSigDB (http://www.gsea‐msigdb.org/gsea/downloads.jsp). GSEA was performed with normalized expression data using python package GSEApy (0.10.2) with the parameters set as follows: cls—class_vector, method—‘signal_to_noise’, min_size—5, max_size—5000. FDR *q*‐values of 0.05 or less were considered significant.

### 2.9 Identification of genes regulated by specific TFs during aging

Transcription factors (TFs) regulate gene expression by binding to promoter regions. We determined the genomic TF footprint using RGT‐HINT, which indicated the locations of the transcription factor binding sites (TFBSs). If the TFBS of a specific TF overlapped with a gene's promoter (±1 kb region around the TSS), the TF was identified as a regulator of that gene.

### 2.10 Immunohistochemistry

Immunohistochemistry was performed using a heat‐mediated antigen retrieval procedure, followed by blocking endogenous peroxidases with hydrogen peroxide. Tissue sections were incubated with a primary antibody overnight at 4°C. Visualization was performed using a diaminobenzidine substrate kit (ZSGB‐Biotech) according to the manufacturer's instructions. An anti‐c‐Jun antibody (Cell Signaling Technology, 9165) and an anti‐PU.1 antibody (Cell Signaling Technology, 2258) were used as the primary antibodies. For quantitation of positive cells, images were acquired by light microscopy (Olympus BX43) at 20× objective magnification. Each slice was measured in at least 6 regions.

### 2.11 Immunofluorescence staining

Mouse tissues were fixed in 4% paraformaldehyde (DingGuo, AR‐0211) for 24 h at room temperature, dehydrated with gradient sucrose solutions (20% and 30% for 24 h each), and embedded in OCT compound (Sakura, 4583) for cryosection. Prior to immunohistochemical staining, the sections (10 μm thick) were fixed in ice‐cold acetone (Xilong Scientific, QC4090) at room temperature for 15 min and blocked with PBS containing 0.3% Triton X‐100 (Sigma‐Aldrich, T8787), 1% normal donkey serum (Jackson ImmunoResearch Laboratories, 017‐000‐121) and 1% bovine serum albumin (Wisent bio products, 800‐095‐QG) at room temperature for 1 h. Samples were incubated with primary antibodies at 4°C overnight, washed three times with PBS, and then incubated with the appropriate secondary antibody for 1 h at 37°C. Nuclei were stained with Hoechst 33342 (Sigma‐Aldrich, B2261). Images were acquired using a ZEISS Axio Scan Z1 (Carl Zeiss) at 20× objective magnification. Each slice was measured in at least 6 regions.

An anti‐c‐Jun antibody (Cell Signaling Technology, #9165), an anti‐PU.1 antibody (Cell Signaling Technology, #2258), and an F4/80 monoclonal antibody (BM8; Invitrogen Antibodies, 14‐4801‐85) were used as the primary antibodies. The secondary antibodies were Alexa Fluor® 488 AffiniPure Donkey Anti‐Rabbit IgG (H + L) (Jackson ImmunoResearch Laboratories, 71‐545‐152), Alexa Fluor® 488 AffiniPure Donkey Anti‐Rat IgG (H + L; Jackson ImmunoResearch Laboratories, 712‐545‐150), Cy™3 AffiniPure Donkey Anti‐Rabbit IgG (H + L; Jackson ImmunoResearch Laboratories, 711‐165‐152), and Goat Anti‐Rat IgG (H + L) Cy3® (Abcam, ab98416).

### 2.12 Flow cytometry analysis

To isolate F4/80^+^ cells from mice kidneys and livers by flow cytometry analysis, the tissues were digested into single‐cell suspension as described (Charni‐Natan & Goldstein, 2020). Cells were then stained with PE anti‐mouse F4/80 (eBioscience, 12–4801‐82) on ice for 1 h and then washed in PBS three times. The cells were suspended in 200 μL of PBS and filtered through a 40 μm nylon cell strainer for analysis. Flow cytometry was performed on an Aria III (BD Biosciences), and the data were analyzed using FlowJo‐V10 (BD).

### 2.13 AP‐1 activity assay

Nuclear protein extracts were isolated following the manufacturer's instructions (Active Motif, 40010). After quantitation using the Bradford assay (Thermfisher, 23246), the 15 μg per sample was added. The AP‐1 subunits (phospho‐c‐Jun) present in active AP‐1 dimers were measured using the ELISA‐based TransAM AP‐1 family (Active Motif, 44296) according to the manufacturer's instructions. The final colorimetric reaction time was 15 min. Absorbance was read using a spectrophotometer (Biotek) within 5 min at 450 nm with a reference wavelength of 655 nm.

### 2.14 SA‐β‐Gal activity assay

SA‐β‐gal staining was performed following the manufacturer's instructions (Beyotime, C0602). Briefly, kidney and liver frozen sections were dried at 37°C for 20 min and fixed in β‐gal fix solution for 15 min at room temperature. After washing in PBS three times, the fixed sections were incubated with β‐gal staining reaction buffer overnight at 37°C without CO_2_. Subsequently, the sections underwent immunofluorescence staining as described in the section 2.11: “Immunofluorescence staining.” Images were randomly acquired using an Inverted Fluorescence Microscope IX73 (Olympus) at a 40× oil objective magnification.

### 2.15 AAV transduction in aged mice

The AAV8 vectors for *in vivo* knockdown were purchased from Azenta. Aged mice were intravenously injected with the AAV8 vectors expressing sh*Fos* or a non‐targeting control vector at a dose of 2 × 10^11^ viral genome particles per mouse via the tail vein using an insulin syringe with a 29‐gauge needle (BD, 320310). The sh*Fos* sequence was as follows: CCGTCTCTAGTGCCAACTTTACTCGAGTAAAGTTGGCACTAGAGACGG.

### 2.16 Reverse transcription‐quantitative PCR (RT‐qPCR)

RNA was reverse‐transcribed to cDNA using TransScript First‐Strand cDNA Synthesis SuperMix (TransGen Biotech, AT311‐03). RT‐qPCR was performed on a CFX96™ Real‐Time System (Bio‐Rad) using Kapa SYBR® FAST qPCR Kit Master Mix (Kapa Biosystems, KM4101). Data were analyzed using the 2^−ΔΔCt^ method. GAPDH was used as a reference gene to normalize the expression of the target genes. The primer sequences used are supplied in Table S1.

### 2.17 Statistics

Data are represented as the mean ± SEM and *p* values were calculated using an unpaired Student's *t*‐test in GraphPad Prism 8 with default parameters. The statistical significance: *p < 0.05, **p < 0.01, ***p < 0.001, ****p < 0.0001.

## 3 RESULTS

### 3.1 Conserved signatures of aging in the kidney and liver as uncovered by transcriptome analysis

**FIGURE 1:**
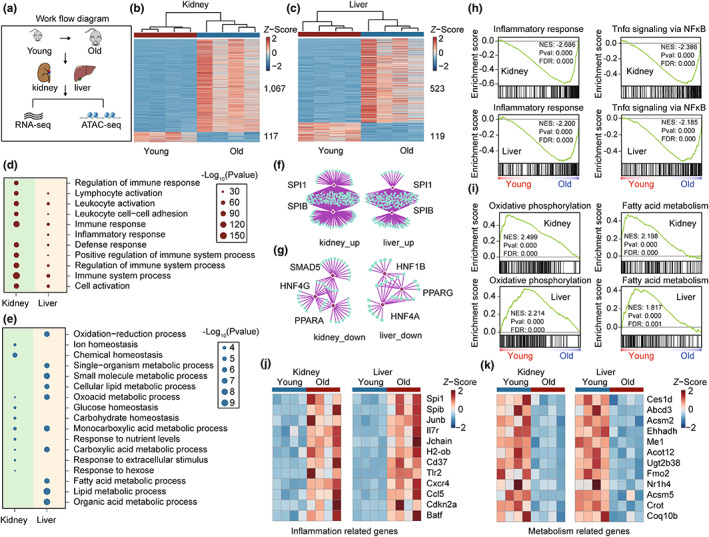
Enhanced inflammation and dysregulated metabolism in the kidney and liver during aging. (a) Schematic outlining the experimental design. The kidney and liver from young (2–3 months) and old (23–25 months) C57BL/6 mice were used for RNA‐seq and ATAC‐seq analysis. (b, c) Heatmap of aging‐associated differentially expressed genes in the kidney (b) and liver (c), (*n* = 4). (d,e) Selected biological process (BP) GO terms enriched in aging‐associated upregulated genes (d) and downregulated genes (e) in the kidney and liver, with the top 10 most significantly enriched terms shown. The size of each point represents the significance of the enrichment. (f,g) Transcription factor enrichment analysis based on aging‐associated upregulated genes (f) and downregulated genes (g) in the kidney and liver using RcisTarget. Only the top significantly enriched TFs are shown. (h,i) GSEA showing changes in inflammation‐related (h) and metabolism‐related (i) pathways in the kidney and liver during aging based on the transcriptome, (*n* = 4). (j,k) Heatmap showing changes in the expression of selected inflammation‐related (j) and metabolism‐related genes (k) in the kidney and liver, (*n* = 4).

To reveal the common signatures and regulatory factors of the complex aging process across tissues, we performed RNA‐seq and ATAC‐seq in the kidney and liver collected from young and aged mice (Figure 1a). Based on RNA‐seq, aging‐associated differentially expressed genes were identified, with 1067 upregulated genes and downregulated 117 genes in the kidney; 523 upregulated genes and 119 downregulated genes in the liver (Figure 1b,c), suggesting an increased transcriptional activity during aging in both tissues. Inflammation‐related genes, including *Cd74*, *Cxcl13*, *Fcrl5*, and *Jchain*, were observed in the set of upregulated genes during aging, while several metabolism‐related genes, including *Pck1*, *Hmgcr*, *Cyp2c50*, and *Mup20*, were found in the set of downregulated genes during aging (Figure S1a,b).

To explore the biological processes affected by aging, we performed gene ontology (GO) analysis based on aging‐associated genes. The GO analysis showed that immune response and inflammation‐related pathways were significantly upregulated in the kidney and liver, while metabolism‐related processes were enriched in the set of aging‐associated downregulated genes, including lipid metabolic process and glucose homeostasis (Figure 1d,e). In agreement with these results, the set of commonly upregulated genes in the kidney and liver was enriched with immune processes, while the set of common downregulated genes was enriched with metabolism‐related processes (Figure S1c,d).

To identify TFs that regulate the transcriptome signatures of aged kidneys and livers, we performed TFs enrichment analysis based on the aging‐associated genes. E26 transformation‐specific (ETS) family TFs, SPI1 and SPIB, were significantly enriched in the set of upregulated aging‐associated genes in the kidney and liver (Figure 1f), which have previously been reported to be involved in the regulation of the immune response (Willis et al., 2017). Moreover, HNFs and PPARs were enriched in the set of downregulated genes in the kidney and liver (Figure 1g), which have previously been demonstrated to play a role in metabolic homeostasis and longevity regulation (Ahmadian et al., 2013; Piedrafita et al., 2021).

To further analyze the signaling pathways that are influenced during aging, we performed gene set enrichment analysis (GSEA). Consistent with the results of GO analysis, GSEA data indicated that inflammatory response and TNF‐α signaling pathways were significantly activated in aged kidney and liver (Figure 1h; Figure S2a). In contrast, metabolism‐related processes, such as oxidative phosphorylation and fatty acid metabolism, were deactivated during aging in both tissues (Figure 1i; Figure S2b). Subsequently, we showed that the gene expression levels of typical inflammation‐related genes, including *Spi1*, *Spib*, *Junb*, and *Ccl5*, were increased in aged kidneys and livers in comparison with those in young tissues, and the expression levels of metabolism‐related genes, such as *Ces1d*, *Acsm2*, and *Coq10b*, were decreased (Figure 1j,k; Figure S3a–d). Collectively, transcriptomics data indicate that enhanced inflammation and dysregulated metabolism are the common signatures of aging between the kidney and liver.

### 3.2 Chromatin accessibility signatures of aging in the kidney and liver as revealed by ATAC‐seq

**FIGURE 2:**
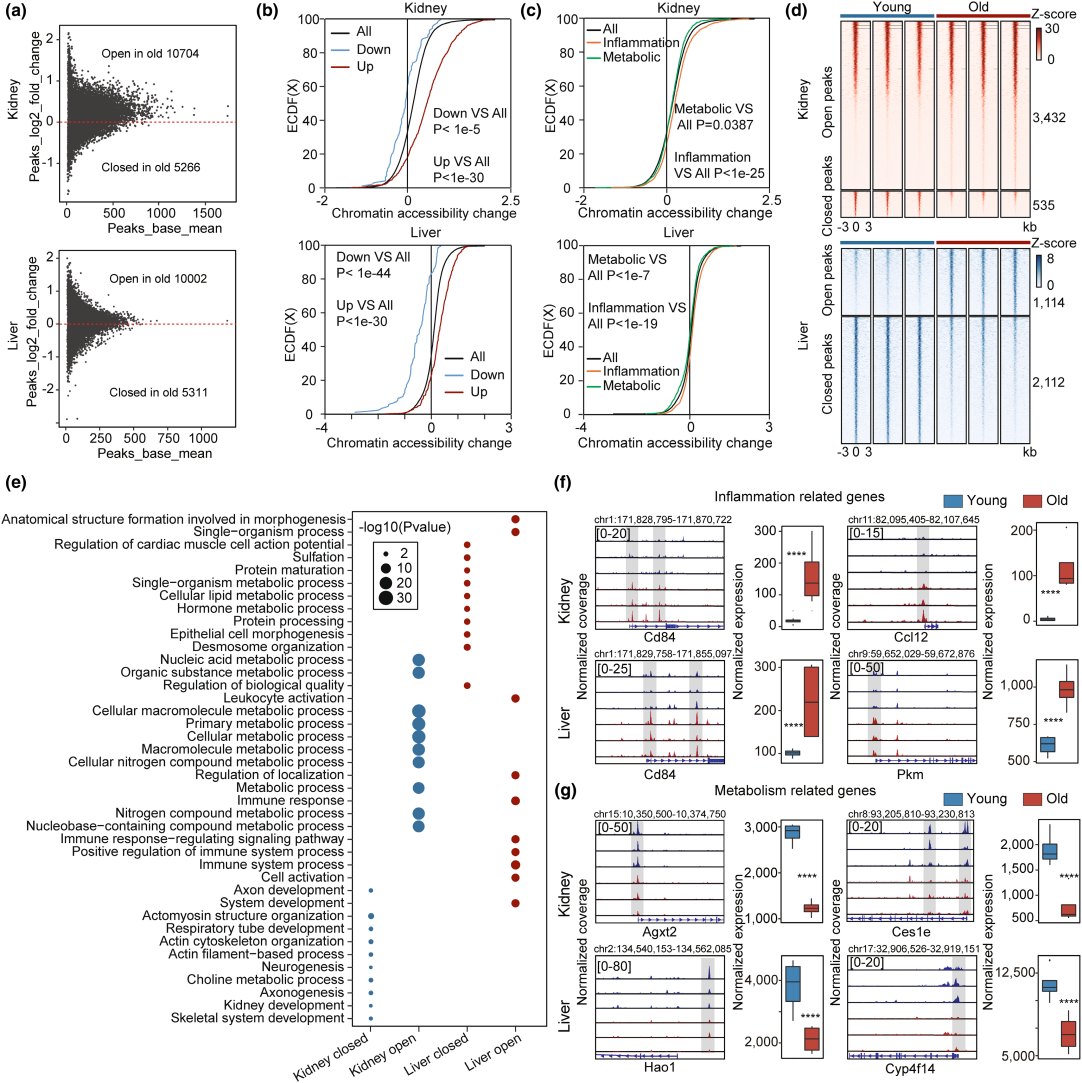
Chromatin accessibility signatures of aging as revealed by ATAC‐seq. (a) MA plot showing the log2 ratio of peak accessibility versus mean normalized read count for peaks located in promoters comparing old and young samples from the kidney (top) and liver (bottom). (b) Cumulative distribution showing the differences in chromatin accessibility between peaks related to upregulated aging‐associated genes and downregulated genes in the kidney (top) and liver (bottom). Peaks associated with upregulated genes showed higher accessibility in comparison with all genes, (*p* < 1e‐30). Peaks associated with downregulated genes showed lower accessibility in comparison with all genes (*p* < 1e‐5 in the kidney; *p* < 1e‐44 in the liver). (c) Cumulative distribution showing the differences in chromatin accessibility between peaks related to metabolism‐related genes and inflammation‐related genes in the kidney (top) and liver (bottom). Peaks associated with metabolism‐related genes showed lower accessibility in comparison with all genes (*p* = 0.0387 in the kidney; *p* < 1e‐7 in the liver). And peaks associated with inflammation‐related genes showed higher accessibility in comparison with all genes (*p* < 1e‐25 in the kidney; *p* < 1e‐19 in the liver). (d) Density heat map showing normalized ATAC‐seq signals ± 3 kb around aging‐associated peaks in the kidney (top) and liver (bottom). The number of peaks within each module is given at the side, (*n* = 3). (e) Biological process GO term enrichment analysis based on genes related to aging‐associated peaks in the kidney and liver, with the top 10 significantly enriched terms shown. The size of each point represents the significance of the enrichment. (f,g) Browser showing changes in the promoter accessibility and expression level of selected genes in the kidney and liver; Browser representation of ATAC‐seq normalized coverage around the transcription start site (TSS) regions of inflammation‐related (f) and metabolism‐related (g) genes. Data were analyzed by unpaired two‐tailed *t*‐test (b,c,f,g). **** *p* < 0.0001.

Regulation of gene expression is always linked to chromatin organization. Previous studies have identified that the gene expression changes are predetermined by the rearrangement of chromatin accessibility (Li et al., 2017; Zhang, Zhang, et al., 2021). To reveal changes in chromatin accessibility that underlie transcriptional changes in the aged kidney and liver, we employed ATAC‐seq, a technology to analyze chromatin accessibility (Buenrostro et al., 2015). Principal component analysis (PCA) based on the ATAC‐seq dataset showed obvious changes in chromatin accessibility in the kidney and liver during aging (Figure S4a). To better visualize the global changes in the kidney and liver during aging, we created MA plots based on the ATAC‐seq data, which demonstrate that chromatin accessibility at promoters increased with aging in the kidney and liver (Figure 2a).

Given the fundamental role of promoter accessibility in gene regulation, we subsequently examined the relationship between promoter accessibility and gene expression during aging. The promoter accessibility of upregulated aging‐associated genes was significantly greater than that of downregulated genes in the kidney and liver, and a strong correlation between promoter accessibility and gene expression was observed (Figure 2b; Figure S4b,c). To examine the epigenetic changes underlying the transcriptional signatures, we analyzed changes in the promoter accessibility of inflammation‐ and metabolism‐related genes collected from MSigDB. In accordance with the transcriptional signatures of aging in the kidney and liver, the promoter accessibility of inflammation‐related genes was greater than the average level in aged kidney and liver, while metabolism‐related genes displayed significantly reduced promoter accessibility (Figure 2c), confirming the epigenetic changes underlying the transcriptional signatures of aging.

To further explore the epigenetic signatures of the aged kidney and liver, we identified aging‐associated peaks using DEseq2. There were 3432 open peaks and 535 closed peaks in the kidney, and 1114 open peaks and 2112 closed peaks in the liver (Figure 2d). And these peaks were found to be evenly distributed across chromosomes (Figure S4d). Interestingly, peak annotation indicated that aging‐associated open peaks were more likely to be located in promoter regions as compared with closed peaks (Figure S4e–j), confirming increased promoter accessibility with aging. Further, GO analysis based on genes related to aging‐associated peaks showed enrichment in immune system and metabolic pathways (Figure 2e).

To elucidate the epigenetic changes underlying dysregulated inflammatory and metabolic processes, we evaluated changes in the promoter accessibility and expression levels of selected genes using Integrative Genomics Viewer (IGV). For example, the inflammation‐related genes *Cd84*, *Ccl12*, and *Pkm* showed dramatically increased expression during aging in the kidney and liver, which were accompanied by increased promoter accessibility (Figure 2f; Figures S5a,b and S6a). In contrast, the metabolism‐related genes *Agxl2*, *Ces1e*, *Hao1*, and *Cyp414* showed decreased promoter accessibility and expression during aging (Figure 2g; Figures S5c,d and S6b), confirming the epigenetic changes underlying aging‐associated transcriptional signatures.

Taken together, ATAC‐seq data show a strong correlation between the transcriptome and epigenome. Importantly, the changes in chromatin accessibility underlying enhanced inflammation and dysregulated metabolism were observed, highlighting the potential role of epigenetic remodeling in the regulation of aging signatures.

### 3.3 Identification of potential regulatory TFs of aging in the kidney and liver

**FIGURE 3:**
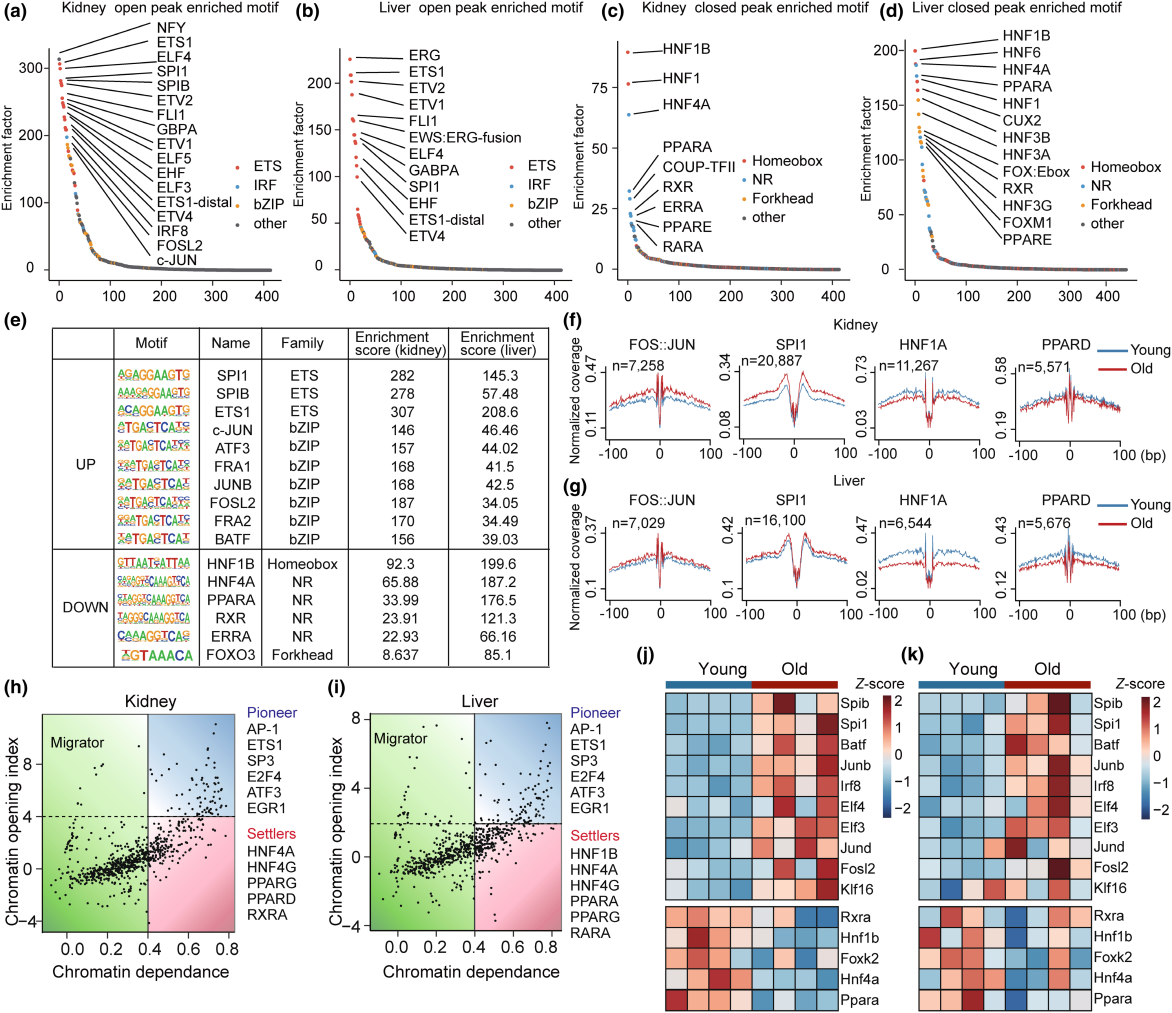
Identification of the regulatory TFs of aging in the kidney and liver. (a–d) Ranking of motifs enriched in aging‐associated open peaks in the kidney (a) and liver (b), and closed peaks in the kidney (c) and liver (d). The colors of the points represent different TF families. (e) Selected commonly enriched TFs in aging‐associated peaks in the kidney and liver. (f,g) TF footprinting in chromatin‐accessible regions based on ATAC‐seq. The mean normalized ATAC‐seq coverage of the forward and reverse strands within 100 bp upstream and downstream of the TF motif is shown. For both the kidney (f) and liver (g), the red line represents young samples and the blue line represents old samples. (h,i) Classification of TFs based on chromatin dependence (CD) and the chromatin opening index (COI). Selected top pioneer factors and settlers in the kidney (h) and liver (i) are shown. (j,k) Heatmap showing changes in the expression levels of identified regulatory TFs with aging in the kidney (j) and liver (k) during aging, (*n* = 4).

Transcription factors have been reported to play important roles in gene regulation and cell fate decisions (Stadhouders et al., 2019). Moreover, previous studies in diverse species and cell types have shown that TFs act as central components of the aging‐regulatory signaling networks (Fischer et al., 2022; Zhou et al., 2018). To identify the regulatory TFs underlying aging in the kidney and liver, we performed motif enrichment analysis based on the aging‐associated peaks and genes described above (Figure 3a–d). Interestingly, we found that the ETS family TFs, SPI1, and SPIB, in addition to the AP‐1 family TFs, c‐JUN, ATF3, and FRA1, were commonly enriched in the open peaks from the kidney and liver (Figure 3e; Figure S7a–d). In contrast, aging‐associated closed peaks from the kidney and liver were commonly enriched in HNFs, PPARs, and RXRs, which is consistent with the transcriptome analyses.

To further explore changes in TF activity during aging, we assessed the relative coverage around TF binding sites using RGT‐HINT footprinting (Li et al., 2019). We identified the footprints of selected TFs described above including c‐JUN, SPI1, HNF1A, and PPARD. Consistent with the results of motif enrichment analysis, c‐JUN, and SPI1 displayed increased activity in the kidney and liver during aging (Figure 3f,g; Figure S8a). In contrast, the activity of HNF1A and PPARD decreased with aging, indicating a potential role for these TFs in the regulation of aging.

Pioneer factors are specific TFs, which induce epigenetic remodeling upon binding (Mayran & Drouin, 2018). Previous work has demonstrated that pioneer factors play an important role in cell fate determination (Soufi et al., 2015). Thus, we speculated that pioneer factors might act as the major regulators of aging signatures. Using PIQ, we classified TFs as pioneer factors, settlers, or migrators based on chromatin dependence (CD) and the chromatin opening index (COI) (Sherwood et al., 2014). AP‐1 and ETS1 were identified as pioneer factors in the aged kidney and liver (Figure 3h,i). Interestingly, metabolism‐related TFs, such as HNFs and PPARs, were identified as settlers, indicating the different regulatory roles of these TFs from the AP‐1, ETS, HNFs, and PPARs families in aging in the kidney and liver.

Next, we examined changes in the expression levels of aging‐associated TFs. TFs that are activated during aging, including AP‐1 and ETS family TFs, showed elevated expression in aged kidneys and livers (Figure 3j,k; Figures S8b and S9). In contrast, TFs that are repressed during aging, including HNFs and PPARs, showed decreased expression with aging in both tissues, indicating a close correlation between the activities and expression levels of these TFs.

**FIGURE 4:**
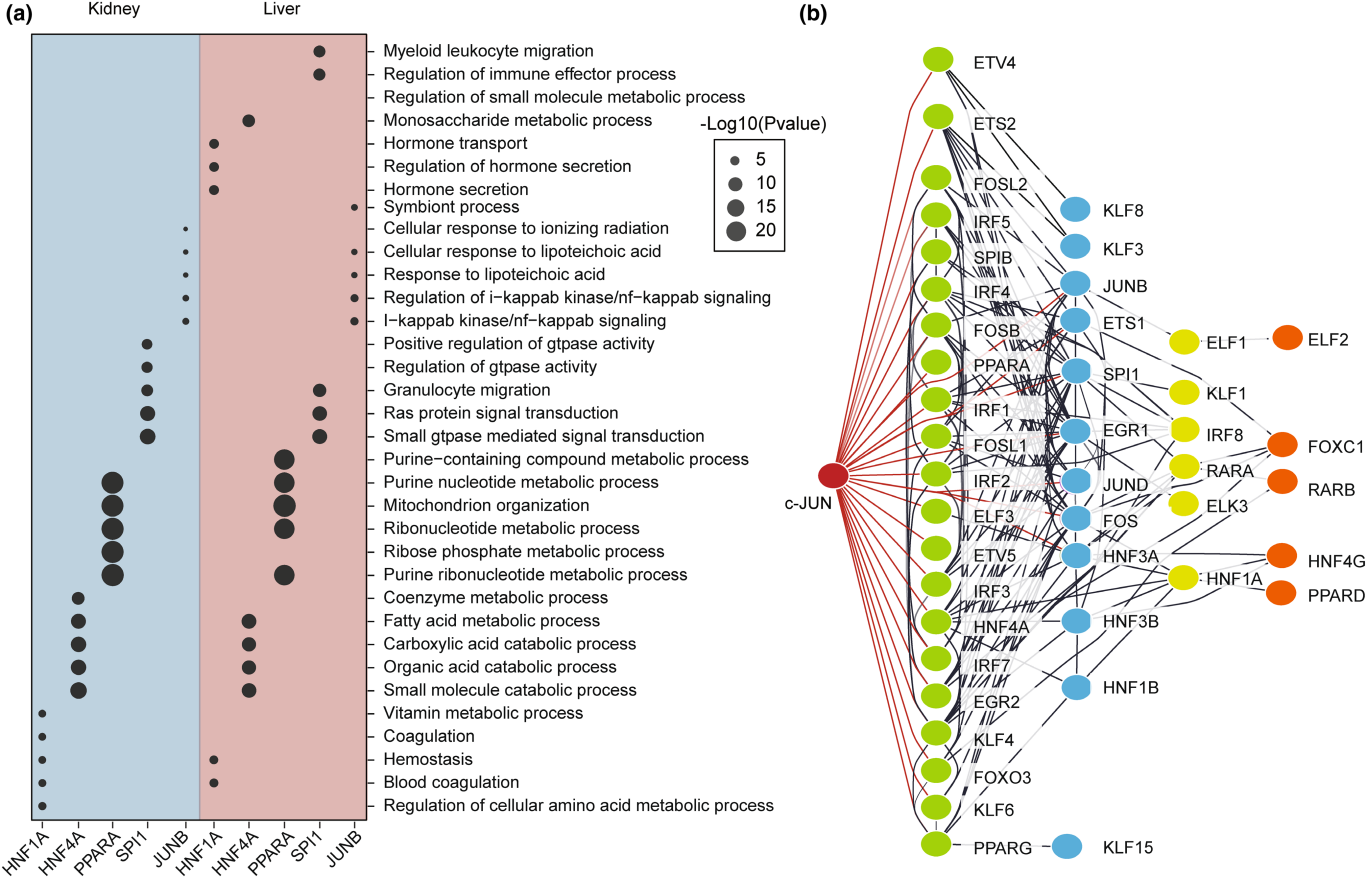
Regulatory network analysis of the identified regulatory TFs. (a) Biological process GO analysis based on TF‐regulated genes. The size of each point represents the significance of the enrichment. (b) Regulatory network analysis based on identified TFs using String. The colors of the TFs represent different layers in the network.

To further explore the regulatory effects of these TFs, we performed GO analysis based on their downstream genes. The GO analysis indicated that HNFs and PPARs regulated metabolism‐related processes in the kidney and liver during aging. In addition, SPI1 and JUNB (a member of the AP‐1 family) regulated inflammation‐related responses (Figure 4a). Subsequently, we carried out regulatory network analysis of aging‐associated TFs using STRING (Szklarczyk et al., 2019). The results of the hierarchy analysis indicated that c‐JUN, a major member of the AP‐1 family, was at the core of the regulatory network, highlighting its potential regulatory role in aging in the kidney and liver (Figure 4b).

Collectively, the results of the regulatory TF analysis indicated that AP‐1 and ETS family TFs act as potential regulators of aging in the kidney and liver, particularly to the enhanced inflammatory response. In addition, the negative regulators HNFs and PPARs showed decreased activity and expression in the kidney and liver, likely contributing to dysregulated metabolism during aging.

### 3.4 The up‐regulation of AP‐1 and ETS in the aged kidney and liver occurs via different mechanisms

**FIGURE 5:**
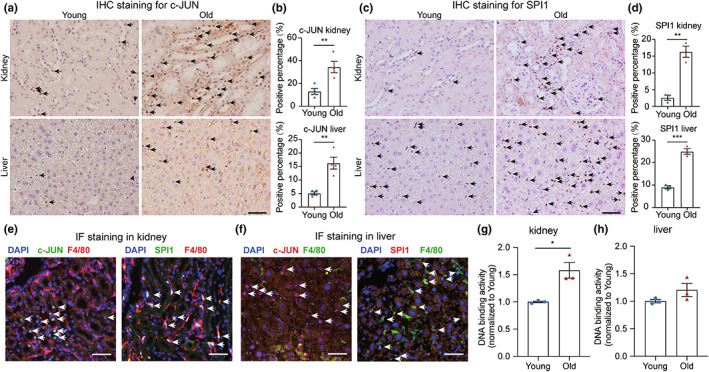
Up‐regulation of AP‐1 and ETS in the aged kidney and liver. (a) IHC staining showing elevated c‐JUN expression in the aged kidney (top) and liver (bottom). c‐JUN^+^ nuclei are labeled with black arrows. Scale bar = 50 μm. (b) Bar plot showing the calculated percentage of c‐JUN^+^ staining in the kidney (top) and liver (bottom), (*n* = 4). (c) IHC staining showing elevated SPI1 expression in the aged kidney (top) and liver (bottom). SPI1^+^ nuclei are labeled with black arrows. Scale bar = 50 μm. (d) Bar plot showing the calculated percentage of SPI1^+^ staining in the kidney (top) and liver (bottom), (*n* = 3). (e) IF staining for nuclei (blue), F4/80 (red), and c‐JUN (green, left panel) or SPI1 (green, right panel) in the aged kidney. c‐JUN^+^ or SPI1^+^ nuclei are labeled with white arrows. Scale bar = 50 μm. (f) IF staining for nuclei (blue), F4/80 (green), and c‐JUN (red, left panel) or SPI1 (red, right panel) in the aged liver. c‐JUN‐ or SPI1‐positive nuclei are labeled with white arrows. Scale bar = 50 μm. (g,h) Normalized DNA‐binding activity of phospho‐c‐Jun in nuclear extracts from the kidneys (g) and livers (h) of young and old mice as measured using a TransAM ELISA kit, (*n* = 3). Data are presented as the means ± SEM. Data were analyzed by unpaired two‐tailed *t*‐test (b,d,g,h). *p < 0.05, **p < 0.01, ***p < 0.001.

To further explore the regulatory mechanism of these potential TFs in aging, we first examined the in situ expression of AP‐1 and ETS in the kidney and liver from young and aged mice by immunohistochemistry (IHC) staining. The IHC staining results showed a significant increase in the expression of c‐JUN and SPI1 in the aged kidney and liver (Figure 5a–d), which was consistent with the changes observed in gene expression (Figure S9). Unexpectedly, we observed that SPI1^+^ cells possessed smaller nuclei and were mostly located in the intercellular space of renal and hepatic cells, while c‐JUN was mainly expressed within renal and hepatic cells. The different locations of c‐JUN and SPI1 in aged kidney and liver suggest diverse mechanisms for these TFs in the regulation of aging.

Previous research has reported that SPI1 plays central role in macrophage maturation and migration (Zakrzewska et al., 2010; Zhang et al., 1994). Furthermore, it has been suggested that chronic inflammation during aging is partly due to the increased infiltration of macrophages into multiple tissues (Wolfe et al., 2018). To further explore whether the enrichment of these TFs during aging is related to the infiltration of macrophages, we performed dual‐staining for c‐JUN or SPI1 and the macrophage marker F4/80 using immunofluorescence (IF) in the aged kidney and liver. The IF staining revealed that c‐JUN was mainly expressed in the nuclei of renal and hepatic cells, while SPI1 was co‐localized with F4/80^+^ cells (Figure 5e,f). Additionally, we performed fluorescence‐activated cell sorting (FACS) to isolate F4/80^+^ and F4/80^−^ cells from tissues. We identified that SPI1 was highly expressed in F4/80^+^ cells compared to other cells and observed a significant increase in the F4/80^+^ cell population in the kidney and liver of old mice relative to young mice (Figure S10). These results suggest that activation of AP‐1 during aging is cellular autonomous, while the activation of SPI1 in aged kidneys and livers is mainly caused by the elevated infiltration of macrophages. Moreover, we quantified the DNA binding activity of c‐JUN in the kidney and liver using a sensitive, non‐radioactive ELISA‐based assay (Challis et al., 2006). The activity of c‐JUN was markedly higher in aged tissues compared to that in young tissues, which is consistent with our footprinting data (Figure 5g,h).

In addition, it is evident that the number of senescent cells increases with age in multiple tissues, leading to the secretion of inflammatory cytokines that generate low‐grade inflammation (Biran et al., 2017; Guerrero et al., 2021). Therefore, we examined whether the increased regions of c‐JUN or SPI1 are associated with the accumulation of senescent cells in aged kidney and liver. The increased lysosome β‐galactosidase (SA‐β‐gal) activity is a commonly used method for assessing the senescence phenotype, both *in vitro* and *in vivo* (Debacq‐Chainiaux et al., 2009; Dimri et al., 1995). Through co‐staining SA‐β‐gal activity with c‐JUN or SPI1 in the kidney and liver, we found that a few cells co‐expressed active SA‐β‐gal and the TFs. And the extent of co‐localization between active SA‐β‐gal and TFs varied depending on the tissue and cell type examined (Figures S11 and S12). These results suggest that the up‐regulation of c‐JUN and SPI1 is associated with cellular senescence in the kidney and liver during aging.

Since the source of inflammatory factors during aging is multifaceted and arises from various cell types in aged tissues (De Maeyer & Chambers, 2021; Mogilenko et al., 2022), the regulation of inflammaging may involve multiple pathways and cellular processes. Based on our findings, the up‐regulation of AP‐1 and SPI1 in aged kidneys and livers may occur through distinct mechanisms, presenting different targets for interventions of inflammaging.

### 3.5 Knockdown of AP‐1 attenuates the inflammatory response in the aged kidney and liver

**FIGURE 6:**
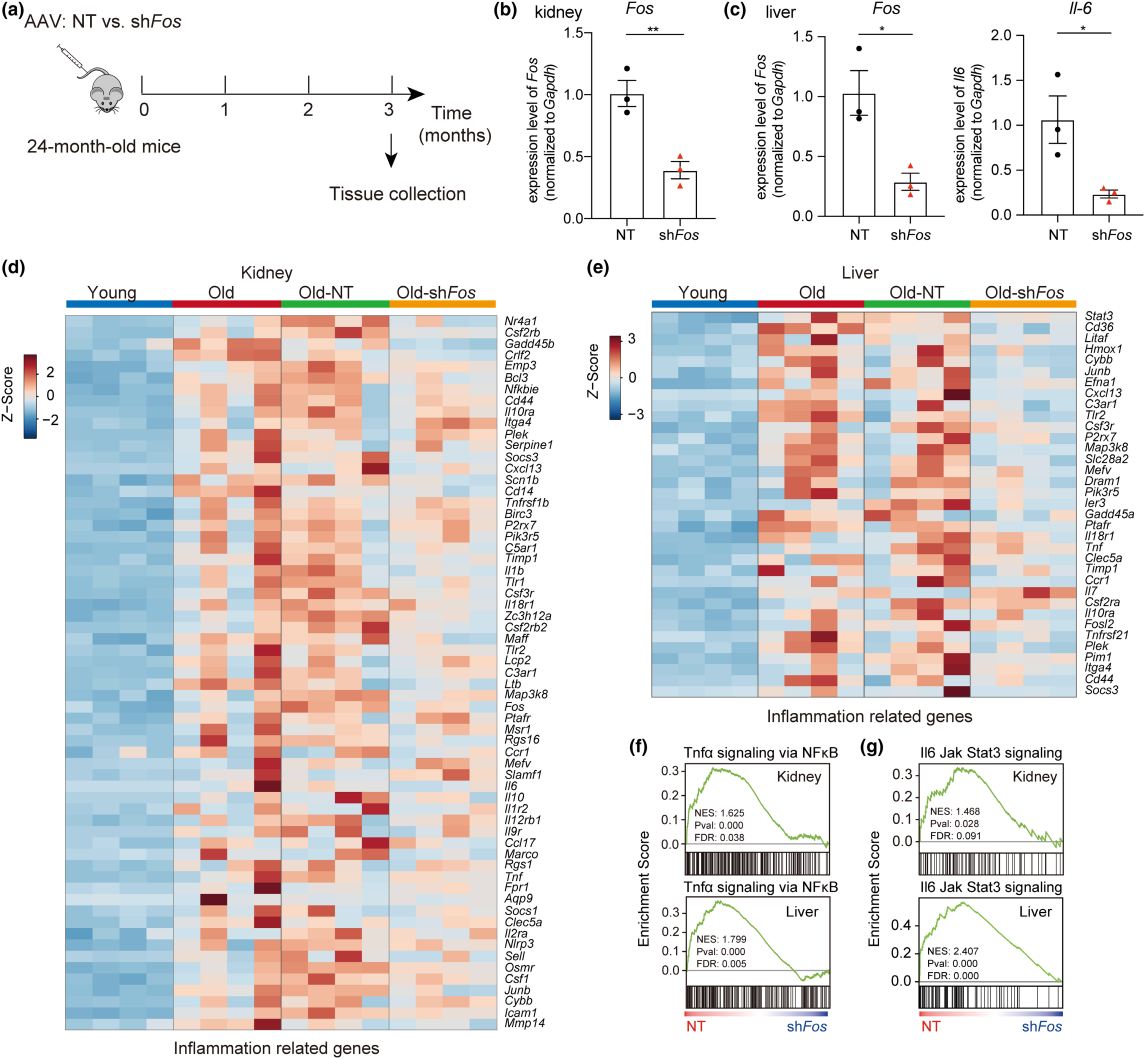
Knockdown of AP‐1 attenuates the inflammatory response in the aged kidney and liver. (a) Experimental design for AAV‐mediated knockdown in aged mice. 24‐month‐old mice were intravenously injected with shFos knockdown or non‐target (NT) AAV‐vectors via the tail vein. (b, c) RT‐qPCR showing the relative gene expression in the kidney (b) and liver (c) of aged mice following Fos knockdown as compared with the control, (*n* = 3). (d, e) GSEA showing the statistically significant gene set: TNF‐α signaling via NF‐κB (d) and Il6‐Jak‐Stat3 signaling (e) were downregulated in the liver (top) and kidney (bottom) of aged mice following Fos knockdown as compared with the control, (*n* = 4). (f, g) Heatmap showing expression changes of inflammation‐related genes in the liver (f) and kidney (g) upregulated in aged mice as compared with young mice, and downregulated in aged mice following Fos knockdown as compared with the control, (*n* = 4). Data are presented as means ± SEM. Data were analyzed by unpaired two‐tailed t‐test (b, c). *p < 0.05, **p < 0.01.

To further explore the functional roles of AP‐1 in the aging process, especially in relation to inflammation, we investigated whether knockdown of AP‐1 could alleviate aging phenotypes in aged mice. We chose to knockdown *Fos*, a major member of the AP‐1 family, which was achieved by intravenous delivery of the adeno‐associated virus (AAV) sh*Fos*‐knockdown vector to 24‐month‐old mice (Figure 6a). The expression of *Fos* was successfully downregulated in the kidney and liver of mice in the sh*Fos* group 3 months post‐intervention as compared with the non‐target (NT) group (Figure 6b,c). In addition, the expression of *Il‐6*, a marker of inflammaging, was also downregulated in the liver of the sh*Fos* group mice (Figure 6c). Further RNA‐seq data revealed that *Fos* knockdown attenuated the expression of inflammation‐related genes in aged livers and kidneys (Figure 6d,e). Moreover, GSEA analysis demonstrated that knockdown of *Fos* significantly downregulated inflammation‐related pathways, including TNF‐α signaling via NF‐κB, and Il6‐Jak‐Stat3 signaling (Figure 6f,g). Taken together, these results suggest that knockdown of AP‐1 attenuates the enhanced inflammatory response in aged mice, highlighting the potential regulatory role of AP‐1 in inflammaging.

## 4 DISCUSSION

Here, we performed transcriptome and epigenome analyses to elucidate the common regulatory mechanism underlying aging in the kidney and liver. Our data reveal a strong correlation between transcriptome changes and chromatin dynamics, demonstrating that enhanced inflammation and dysregulated metabolism are common signatures of aging in the kidney and liver at both the transcriptional and epigenetic levels. More importantly, we identified the AP‐1, ETS, HNFs, and PPARs families as potential regulatory TFs of aging in the kidney and liver. Moreover, the genetic manipulation of AP‐1 attenuated the inflammatory response in both tissues of aged mice, suggesting that AP‐1 is an upstream regulator of inflammaging.

Dynamic epigenetic networks are a crucial component of aging, which has been reinforced by the emergence of epigenetic clocks based on DNA methylation and chromatin accessibility (Field et al., 2018; Horvath, 2013; Rechsteiner et al., 2022). However, the chromatin dynamics during aging in mouse tissues remain to be explored. Our study reveals increased promoter accessibility in the kidney and liver (Figure 2a). Based on the “loss of heterochromatin” theory of aging, an increase in chromatin accessibility indicates that these regions become derepressed, leading to aberrant gene expression patterns (Tsurumi & Li, 2012). Global chromatin accessibility profiling has demonstrated that aged satellite cells exhibit a more open chromatin state, causing chronic activation from a quiescent state and dysfunction in regeneration (Dong et al., 2022). In the present study, we observed a dramatic increase in promoter accessibility of inflammation‐related genes in the kidney and liver, as well as a decrease in promoter accessibility of metabolism‐related genes (Figure 2c,f,g). Moreover, the chromatin dynamics showed a strong correlation with gene expression changes, indicating that epigenetic remodeling may be the underlying cause of aging signatures.

Previous studies have highlighted tissue‐specific signatures when comparing cross‐tissue transcriptomes (Schaum et al., 2020; Srivastava et al., 2020). Although tissues age differently, it is of interest to identify aging‐related signatures shared across tissues. Here, we demonstrate that enhanced inflammation and dysregulated metabolism are common transcriptional signatures between the kidney and liver during aging (Figure 1d,e,h,i), and for the first time reveal common epigenetic signatures underlying these transcriptional changes in two tissues. Given the systematic nature of inflammaging, it is reasonable that the upregulation of inflammatory factors during aging can affect multiple tissues, including the kidney and liver. From a clinical perspective, it is noteworthy that the levels of inflammatory cytokines have been quantitated for diagnosis of aging‐related diseases (Liu et al., 2021; O'Brown et al., 2015). Moreover, aging induces the dysregulation of glucose and lipid metabolism, leading to a reduction in the ability to maintain functional homeostasis in multiple tissues (Catic, 2018). Metabolomic analysis of mouse tissues has shown that aging‐associated metabolic footprints can be used as universal biomarkers of aging (Zhang, Kerbl‐Knapp, et al., 2021). Our study demonstrates common signatures in the kidney and liver at the molecular level during aging, suggesting their use as robust tissue‐independent hallmarks of aging.

More importantly, we identified AP‐1, ETS, HNFs, and PPARs as commonly enriched TFs based on aging‐associated genes and peaks (Figures 1f,g, 3e, and 4a), among which AP‐1 and ETS family TFs act as positive regulators of inflammation during aging, and HNFs and PPARs family TFs act as negative regulators, likely contributing to dysregulated metabolism. Specifically, AP‐1 and ETS family TFs have been reported to be involved in immunity and inflammation (Pimenova et al., 2021; Qiao et al., 2016; Schonthaler et al., 2011). AP‐1 controls diverse cellular process, including proliferation, apoptosis, differentiation, and transformation (Ye et al., 2014). In addition, it is reported to regulate the senescence program in oncogene‐induced senescent cells (Martinez‐Zamudio et al., 2020). ETS TFs are conserved in animals and generally function as transcriptional activators (Sharrocks, 2001). ETS1 has recently been linked to longevity via its ribosome‐inactivating function (Xiao et al., 2022). In addition, knockdown of the ETS1 homolog and activation of its transcriptional repressor can extend lifespan in Drosophila (Alic et al., 2014; Dobson et al., 2019). Moreover, PPARs and HNFs have been shown to play important roles in the regulation of metabolic homeostasis in the kidney and liver (Piedrafita et al., 2021; Wang, 2010). PPARs are critical regulatory TFs of glucose and lipid metabolism (Ahmadian et al., 2013). It has been demonstrated that activation of PPARγ improves insulin sensitivity and extends longevity in mice (Xu et al., 2020). HNFs act as key regulators of lipid metabolic homeostasis (Liu et al., 2022), although their possible implication in aging has not been extensively studied. A multi‐tissue aging‐related gene expression signature in rat also shows enrichment of the HNFs motif in the aging kidney (Shavlakadze et al., 2019). Taken together, these data indicate that the above‐mentioned TFs may serve as the candidate aging regulators of aging, and further studies will deepen our understanding of aging mechanisms.

Further, we found that AP‐1 and ETS family TFs may have distinct regulatory mechanisms in inflammaging, since enrichment of SPI1 in the kidney and liver was mainly caused by the infiltration of immune cells (Figure 5e,f). We surmise that the activation of SPI1 in the aged kidney and liver is associated with immunosenescence, during which macrophages are the predominant immune cell type and release inflammatory cytokines (De Maeyer & Chambers, 2021). Notably, AP‐1 was autonomously upregulated in renal and hepatic cells during aging and identified as the pioneer factor at the top of the TF network hierarchy (Figures 3h,i, and 4b). The AP‐1 family is composed of Jun, Fos, ATF, and MAF family proteins, which function as dimers (Karin et al., 1997). Other groups have demonstrated that the AP‐1 family proteins, such as c‐Jun and ATF3, play functional roles in different models of cellular senescence (Martinez‐Zamudio et al., 2020; Wang, Liu, Song, et al., 2022; Zhang, Zhang, et al., 2021). Our study validated AP‐1 function *in vivo* as a manipulator of the enhanced inflammatory response in the kidney and liver of aged mice (Figure 6). Knockdown of c‐fos, the major member of the AP‐1 family, had positive effects on aging, while knockdown of c‐Jun caused increased mortality and tumorigenesis (data not shown). Although c‐Jun and c‐fos can form heterodimers (Ciapponi & Bohmann, 2002), these proteins may play independent roles in aging. Considering these data together, we propose that AP‐1 is a potential regulator of inflammaging; however, detailed mechanisms, such as specific chromatin marks and interaction networks affected by AP‐1, should be studied further.

In summary, our results reveal the common signatures of aging in the kidney and liver at the transcriptome and epigenome levels, as well as the common regulatory TFs underlying these aging signatures. In addition, we identified AP‐1 as a potential regulatory TF of chronic inflammation during aging, which may provide a novel target for the treatment of aging‐related chronic diseases.

## AUTHOR CONTRIBUTIONS

X.Y. and Y.W. designed the experiments, performed the bioinformatics analysis, and wrote the manuscript. Y.W. prepared ATAC‐seq library. X.Y. performed immunostaining and tissue section analyses. X.Y. and Y.S. performed animal experiments. X.G. participated in the tissue collection. H.D. supervised the project.

## FUNDING INFORMATION

This work is supported by the National Natural Science Foundation of China (32288102).

## CONFLICT OF INTEREST STATEMENT

The authors declare no competing interests.

## CODE AVAILABILITY

All the custom codes written in Python or R are available from the corresponding authors upon reasonable request.

## Supporting information


Figures S1‐S12

Table S1

## Data Availability

All the high‐throughput sequencing data generated in this study have been deposited in the GEO database under the accession number GSE181797 and GSE221801. All other data supporting our findings in this study are available from the corresponding authors upon reasonable request.
