# Supplementary material for: AP‐1 is a regulatory transcription factor of inflammaging in the murine kidney and liver
Source: Aging Cell. 2023 May 8;22(7):e13858. doi: 10.1111/acel.13858 (PMC10352569; doi:10.1111/acel.13858)
Supplement: Supplementary file 1 — Figures S1‐S12 [file ACEL-22-e13858-s002.pdf]

Supplementary Figure 1

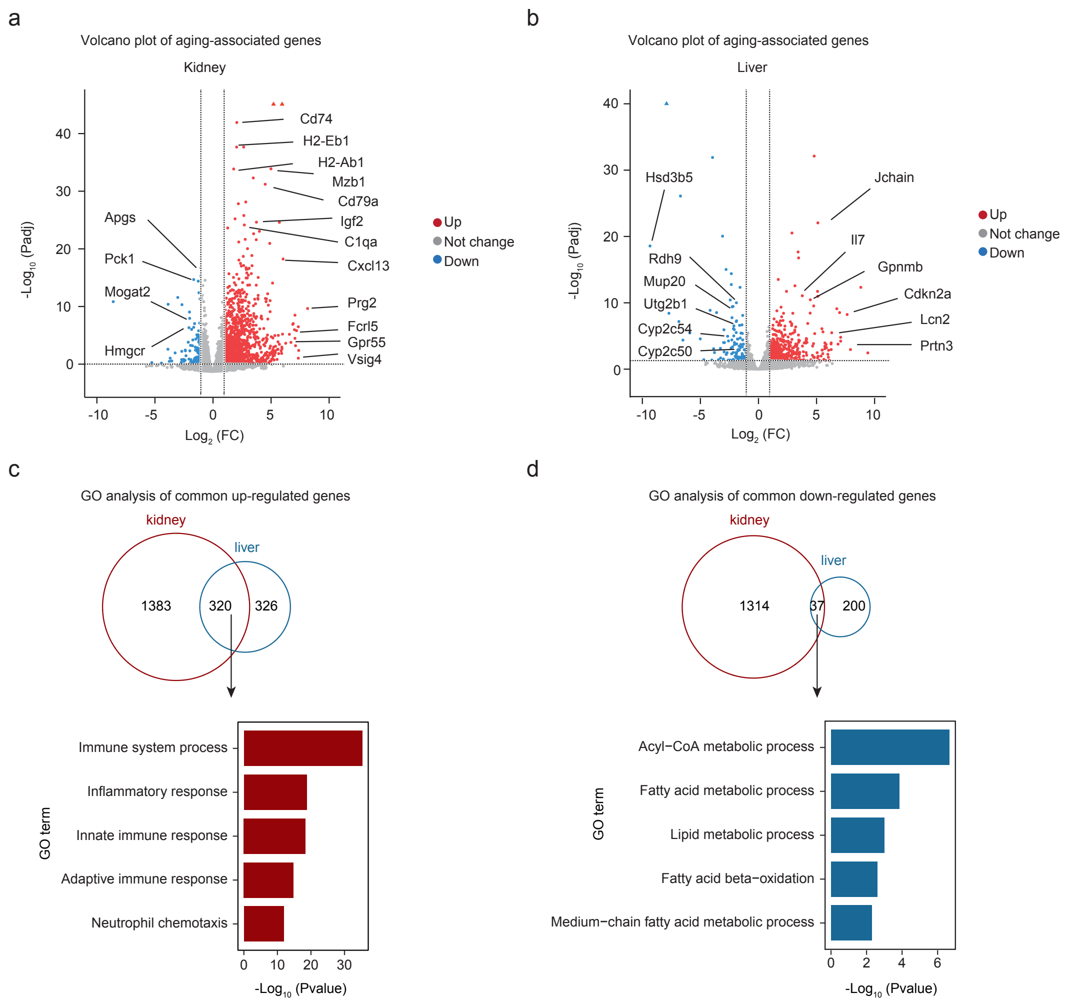

Supplementary Figure 1. Transcriptional signatures of aging in the kidney and liver.

(a, b) Volcano plot showing aging-associated genes in kidney (a) and liver (b). Blue dots represent aging-associated downregulated genes, and red dots represent aging-associated upregulated genes. And selected typical inflammation and metabolism related genes are labeled.

(c, d) Biological process GO analysis based on commonly significantly changed genes in kidney and liver. Top 5 most significantly enriched terms in commonly upregulated genes (c) and downregulated genes (d) are shown.

Supplementary Figure 2

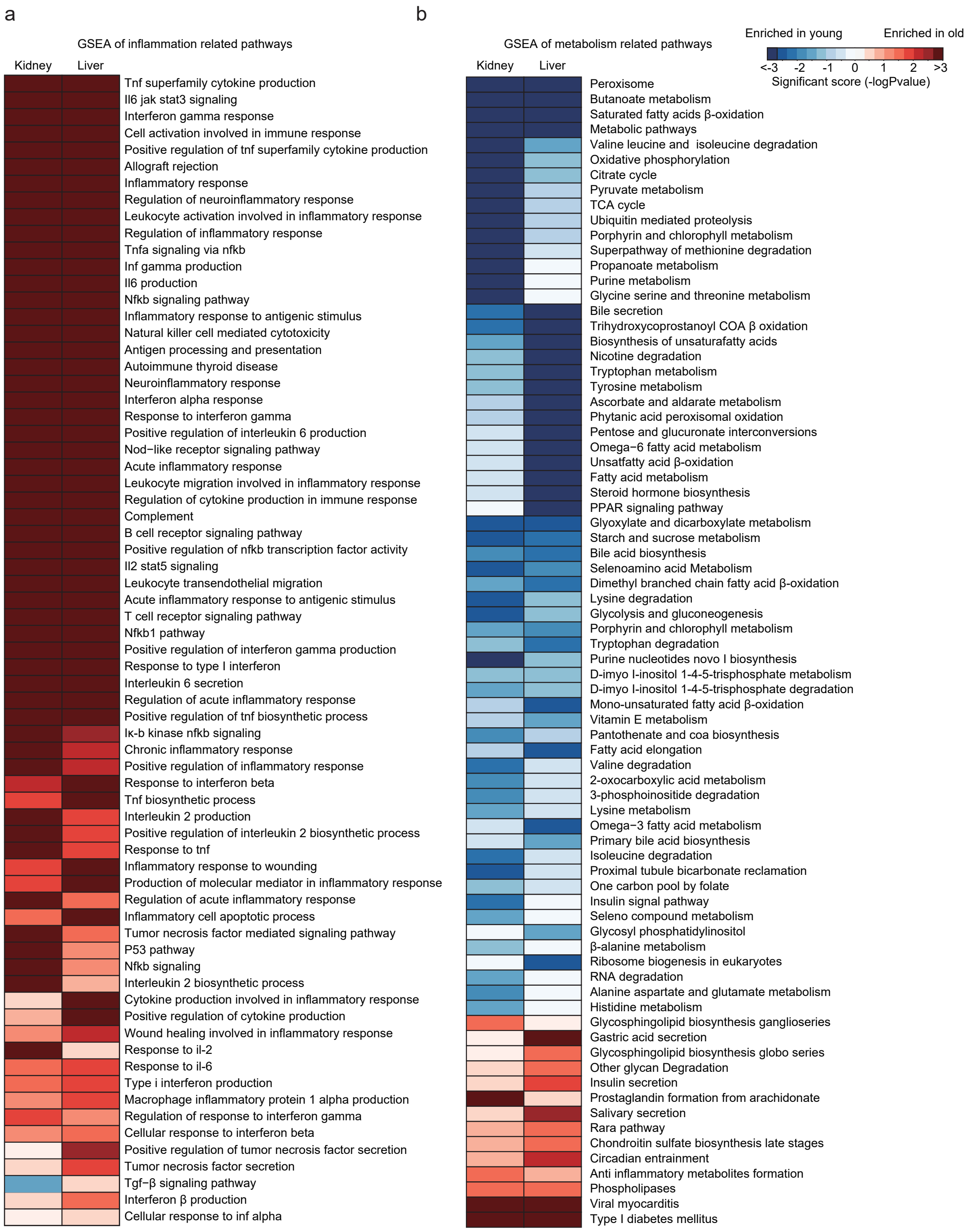

Supplementary Figure 2. GSEA analysis of transcriptional signatures in the kidney and liver during aging.

(a, b) Heatmap showing GSEA analysis based on transcriptional changes with aging in kidney and liver for metabolism related terms (a) and inflammation related terms (b). The color represents enrichment significance in aged mice. Blue color represents down-regulated terms with aging, and red color represents up-regulated terms with aging.

Supplementary Figure 3

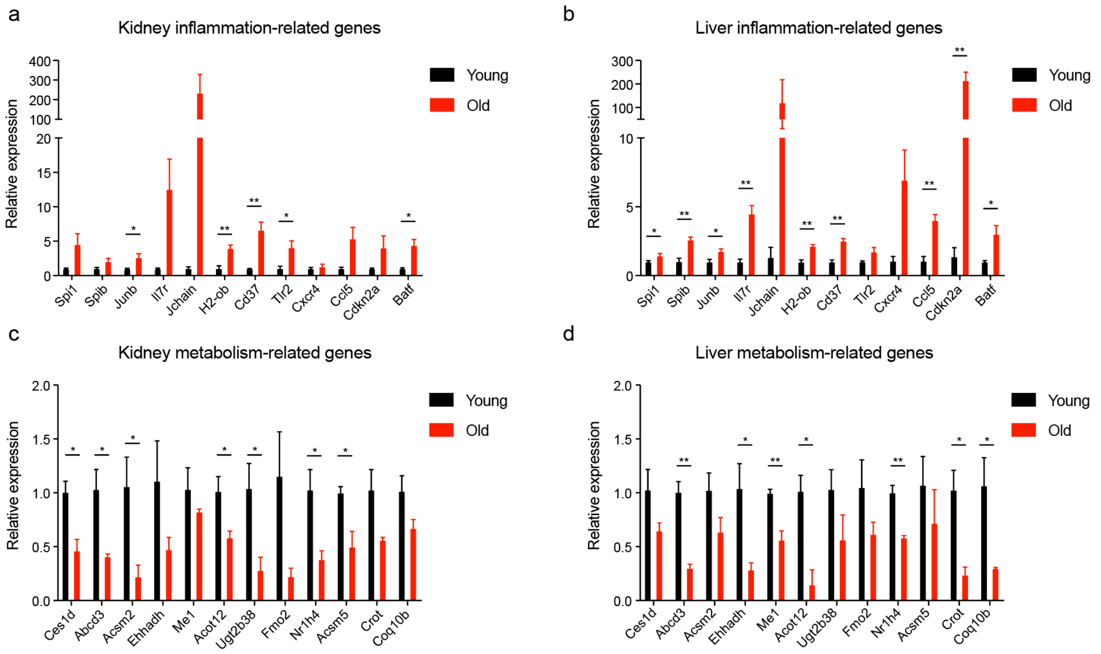

Supplementary Figure 3. RT-qPCR analysis of typical inflammation-related genes and metabolism-related genes in the kidney and liver.

(a, b) RT-qPCR showing the relative expression of inflammation-related genes in the kidney (a) and liver (b) of young and old mice, (n = 3).

(c, d) RT-qPCR showing the relative expression of metabolism-related genes in the kidney (c) and liver (d) of young and old mice, (n = 3).

Data are presented as means ± S.E.M. Data were analyzed by unpaired two-tailed *t*-test (a–d). * $P < 0.05$, ** $P < 0.01$.

Supplementary Figure 4

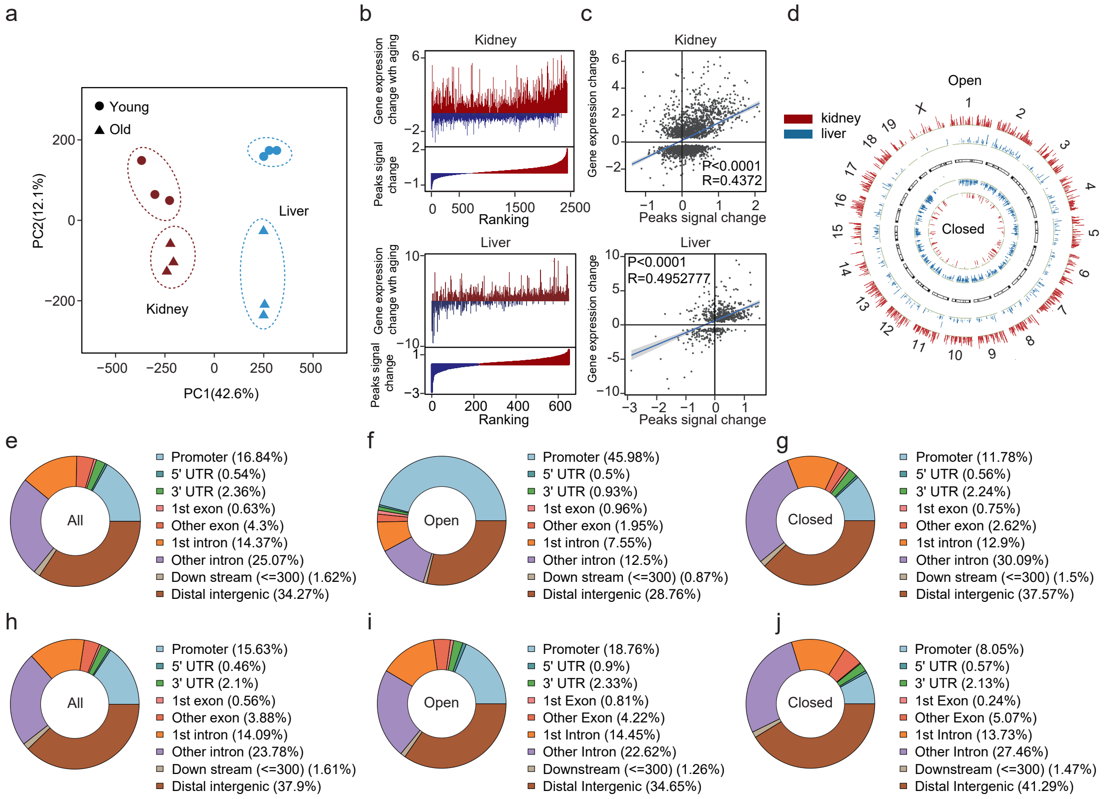

Supplementary Figure 4. Epigenetic signatures of aged kidney and liver revealed by ATAC-seq.

(a) PCA of chromatin accessibility detected with ATAC-seq in the kidney (dark red) and liver (blue).

(b, c) Ranking bar plot and scatter plot showing the correlation between promoter accessibility and gene expression in the kidney (b) and liver (c). Red bar represents upregulated peaks and genes in aged tissues, and blue bar represents downregulated peaks and genes in aged tissues.

(d) Circular plot showing distribution and accessibility of aging-associated peaks in the kidney (red), and liver (blue) on genome.

(e–j) Pie charts showing the proportions of distribution on genome for aging associated peaks. All peaks (e), open peaks (f) and closed peaks (g) in the kidney, and all peaks (h), open peaks (i) and closed peaks (j) in the liver are analyzed individually.

Supplementary Figure 5

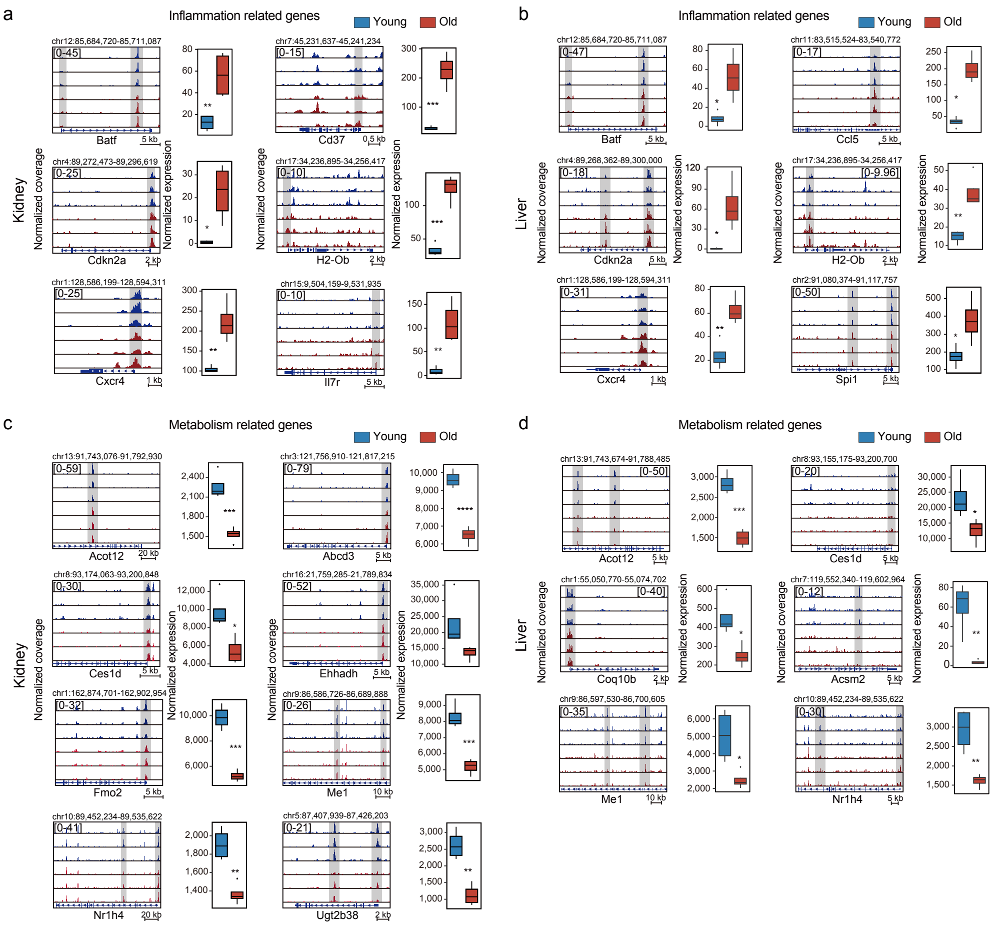

Supplementary Figure 5. Genome browser visualization of typical genes in the kidney and liver.

(a, b) Browser showing changes in the promoter accessibility and expression level of inflammation-related genes in the kidney (a) and liver (b);

(c, d) Browser showing changes in the promoter accessibility and expression level of metabolism-related genes in the kidney (c) and liver (d).

Data were analyzed by unpaired two-tailed *t*-test (a–d). * $P < 0.05$, ** $P < 0.01$, *** $P < 0.001$.

Supplementary Figure 6

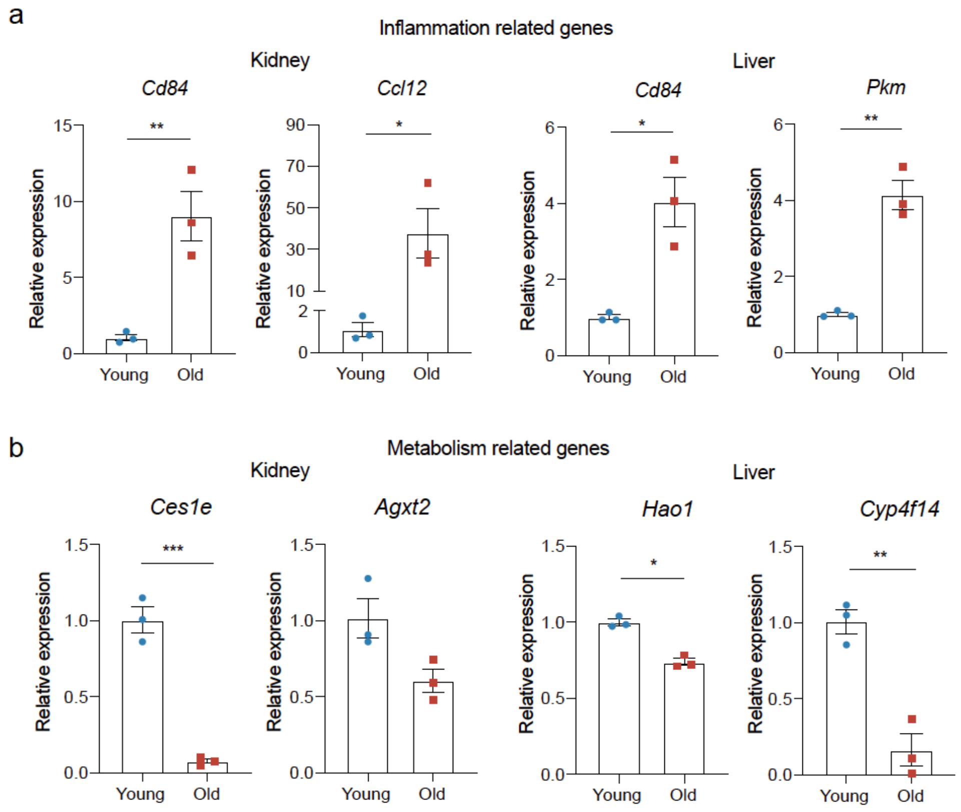

Supplementary Figure 6. RT-qPCR analysis of selected genes showed in Genome Browser

(a, b) RT-qPCR showing the relative expression of selected inflammation (a) and metabolism (b) related genes showed in Genome Browser in the kidney (left) and liver (right) of young and old mice, (n = 3).

Data are presented as means ± S.E.M. Data were analyzed by unpaired two-tailed *t*-test (a, b). * $P < 0.05$, ** $P < 0.01$, *** $P < 0.001$.

Supplementary Figure 7

a

Commonly Enriched motifs in aging-associated open peaks

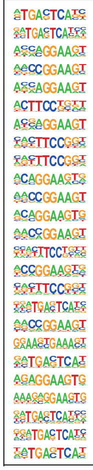

Motif	Name	Family	Enrichment score (kidney)	Enrichment score (liver)
	AP-1	bZIP	146	46.46
	ATF3	bZIP	157	44.02
	EHF	ETS	224	144.5
	ELF1	ETS	177	65.34
	ELF3	ETS	212	121.1
	ELF4	ETS	300	160.5
	ELF5	ETS	243	112.1
	ELK1	ETS	144	49.21
	ELK4	ETS	147	55.48
	ERG	ETS	228	225.7
	ETS	ETS	149	59.19
	ETS1	ETS	307	208.6
	ETV1	ETS	246	202.4
	ETV2	ETS	275	208.5
	ETV4	ETS	209	135.9
	FLI1	ETS	256	188
	FRA1	bZIP	168	41.5
	GABPA	ETS	249	160.4
	IRF8	IRF	198	45.47
	JUNB	bZIP	168	42.57
	SPI1	ETS	282	145.3
	SPIB	ETS	278	57.48
	FOSL2	bZIP	187	34.05
	FRA2	bZIP	170	34.49
	BATF	bZIP	156	39.03

b

Commonly Enriched motifs in aging-associated closed peaks

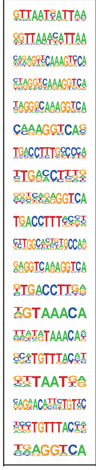

Motif	Name	Family	Enrichment score (kidney)	Enrichment score (liver)
	HNF1B	Homeobox	92.3	199.6
	HNF1	Homeobox	81.61	172.3
	HNF4A	NR	65.88	187.2
	PPARA	NR	33.99	176.5
	RXR	NR	23.91	121.3
	ERRA	NR	22.93	66.16
	PPARE	NR	18.89	112.2
	RARA	NR	18.63	64.18
	EAR2	NR	17.19	72.49
	NUR77	NR	15.97	62.47
	NF1	CTF	12.84	21.08
	TR4	NR	12.51	48.95
	ESRRB	NR	9.992	35.32
	FOXO3	Forkhead	8.637	85.1
	FOXF1	Forkhead	8.552	58.96
	FOXK2	Forkhead	7.469	59.94
	NKX6.1	Homeobox	6.501	17.86
	GRE	NR	6.221	11.64
	FOXP1	Forkhead	5.299	63.84
	THRB	NR	5.239	37.89

c

Commonly Enriched motifs in aging-associated up-regulated genes

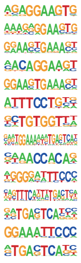

Motif	Name	Family	Enrichment score (kidney)	Enrichment score (liver)
	SPI1	ETS	31.74	14.15
	SPIB	ETS	30.14	12.29
	IRF8	IRF	24.87	3.474
	ETS1-DISTAL	ETS	23.46	7.563
	SPI1:IRF8	ETS	22.5	3.591
	EWS:ERG	ETS	20.06	5.668
	RUNX-AML	Runt	11.99	5.307
	NFAT:AP1	RHD	11.86	3.667
	RUNX	Runt	11.05	3.362
	NFKB-P65	RHD	8.797	3.331
	BZIP:IRF	bZIP	7.111	4.485
	JUN-AP1	bZIP	6.088	5.501
	NFKB-P65-REL	RHD	5.889	5.435
	AP-1	bZIP	5.289	6.575

d

Commonly Enriched motifs in aging-associated down-regulated genes

Motif	Name	Family	Enrichment score (kidney)	Enrichment score (liver)
	HNF4A	NR	9.338	12.49

Supplementary Figure 7. Commonly enriched TFs in the kidney and liver during aging based on ATAC-seq and RNA-seq.

(a, b) Commonly enriched motifs in aging-associated peaks in the kidney and liver. Commonly significantly enriched motifs in open peaks (a) and closed peaks (b) are shown.

(c, d) Commonly enriched motifs in aging-associated genes in the kidney and liver. Commonly significantly enriched motifs in up-regulated genes (a) and down-regulated genes (b) are shown. Motif analysis is performed using HOMER.

Supplementary Figure 8

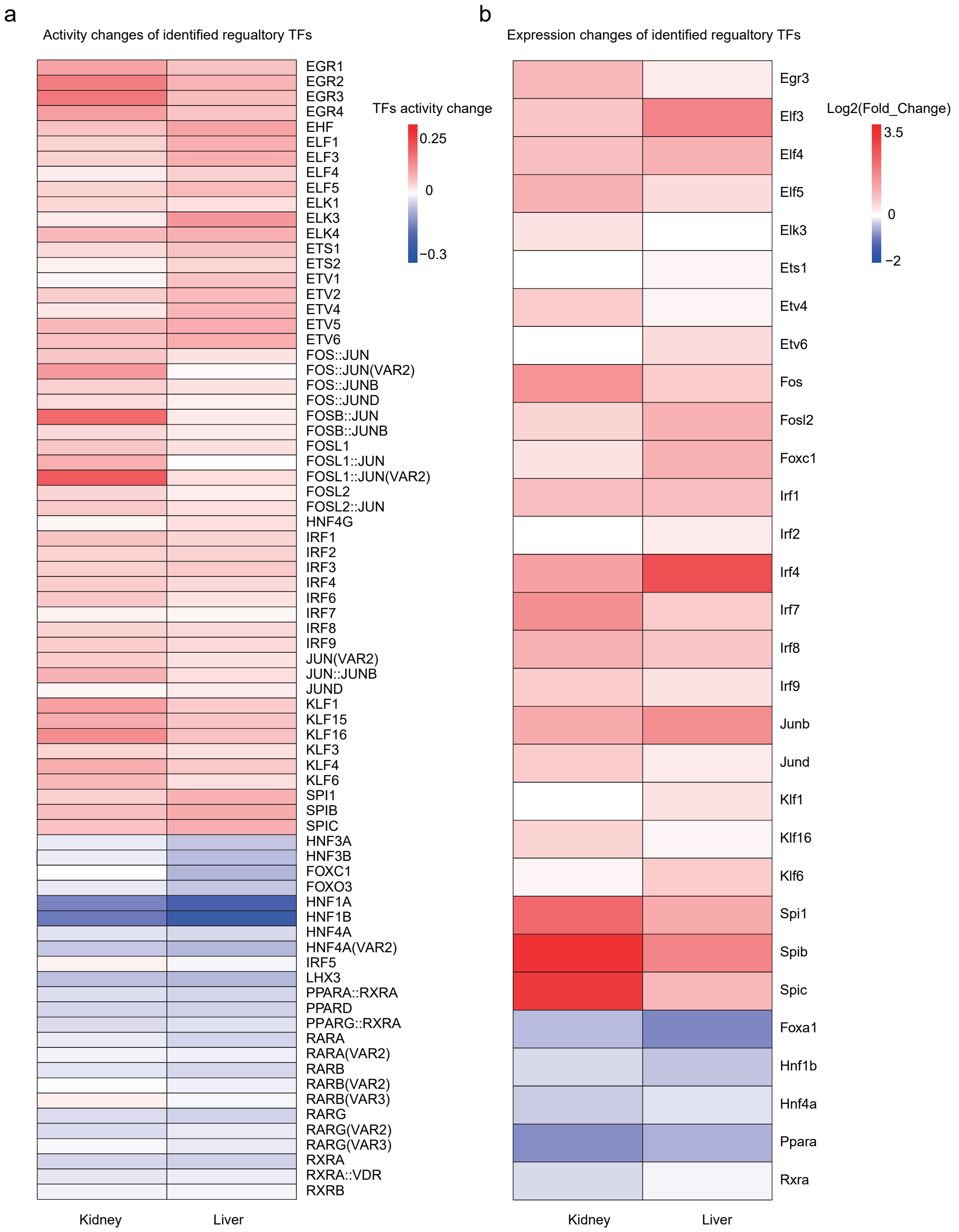

Supplementary Figure 8. Activities and expression changes of regulatory TFs in the kidney and liver during aging.

(a) Heatmap showing the activity changes of identified potential regulatory TFs in the kidney and liver during aging. The color represents activity changes during aging. Red represents increased activities and blue represents decreased activities during aging.

(b) Heatmap showing the expression changes of selected TFs in the kidney and liver during aging. The color represents gene expression changes during aging. Red represents increased gene expression and blue represents decreased gene expression during aging.

Supplementary Figure 9

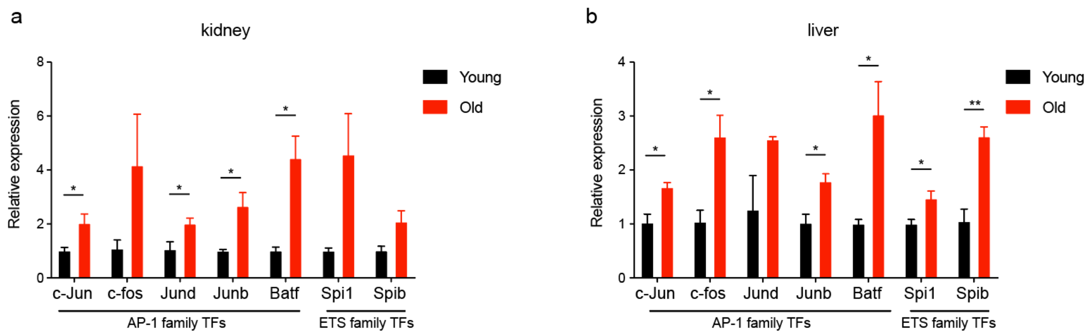

Supplementary Figure 9. RT-qPCR analysis of AP-1 and ETS family TFs in the kidney and liver

(a, b) RT-qPCR showing the relative gene expression of AP-1 and ETS family TFs in the kidney (a) and liver (b) of young and old mice, (n = 3).

Data are presented as means ± S.E.M. Data were analyzed by unpaired two-tailed *t*-test (a, b). * $P < 0.05$, ** $P < 0.01$.

Supplementary Figure 10

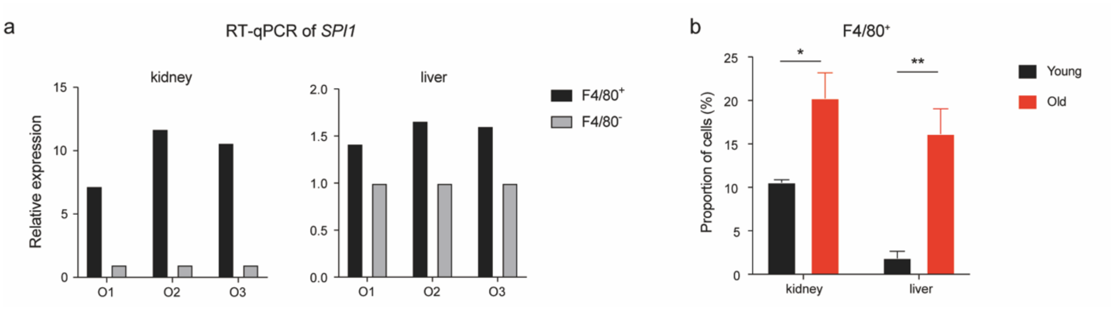

Response Figure 10. Validation of SPI1 in the kidney and liver by FACS

(a) RT-qPCR showing the relative gene expression of *SPI1* in sorted F4/80$^+$ cell as compared to F4/80$^-$ cells in the kidney (left) and liver (right) of aged mice. O1, O2, and O3 represents different mice.

(b) Quantification of the proportion of F4/80$^+$ cells in the kidney and liver of young and old mice, (n=3).

Data are presented as means ± S.E.M. Data were analyzed by unpaired two-tailed *t*-test (a, b). * $P < 0.05$, ** $P < 0.01$.

Supplementary Figure 11

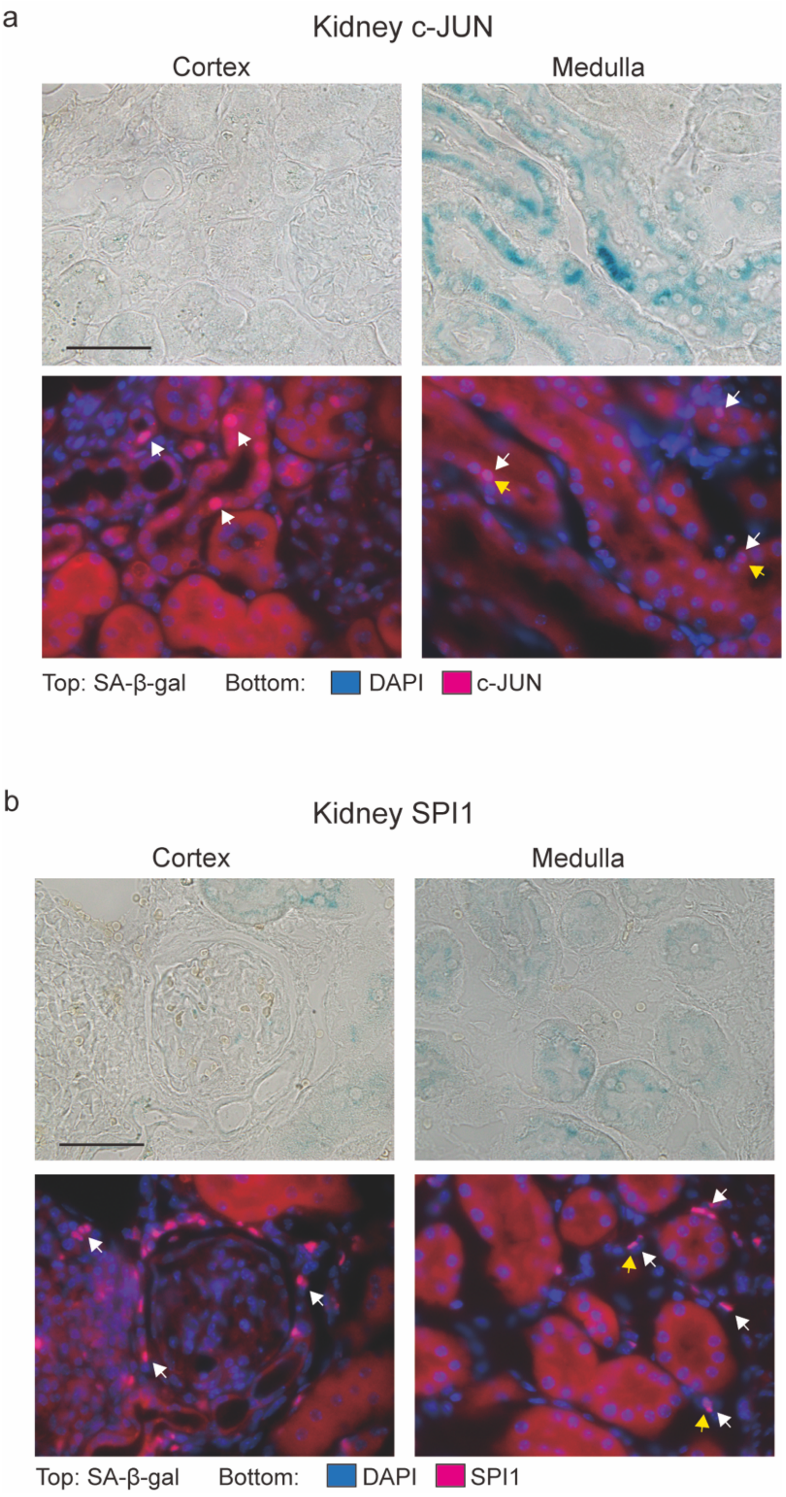

Supplementary Figure 11. The detection of cellular senescence in aged kidney.

(a) Activation of c-JUN in senescence cells was examined by co-staining for SA-β-Gal activity (top) and c-JUN by immunofluorescence (bottom) in aged kidney. SA-β-gal^{+} cells were predominantly observed in the tubular epithelium, distributing throughout both the renal cortex and medulla. c-JUN was observed to be expressed in the nuclei of renal tubal epithelial cells, in both the cortex and the medullar. And some of the c-Jun^{+} cells co-expressed SA-β-gal activity.

(b) Activation of SPI1 in senescence cells was examined by co-staining for SA-β-Gal activity (top) and SPI1 by immunofluorescence (bottom) in aged kidney. SPI1 was localized in irregular nuclei located in the space between renal tubules or around the glomerulus. And some of the $SPI1^+$ cells exhibited weak SA-β-gal activity.

The regions of the renal cortex and the medullar were randomly photographed. And $c\text{-}JUN^+$ or $SPI1^+$ nuclei are labeled with white arrows and active SA-β-gal nuclei are labeled with yellow arrows. Scale bar = 50 μm.

Supplementary Figure 12

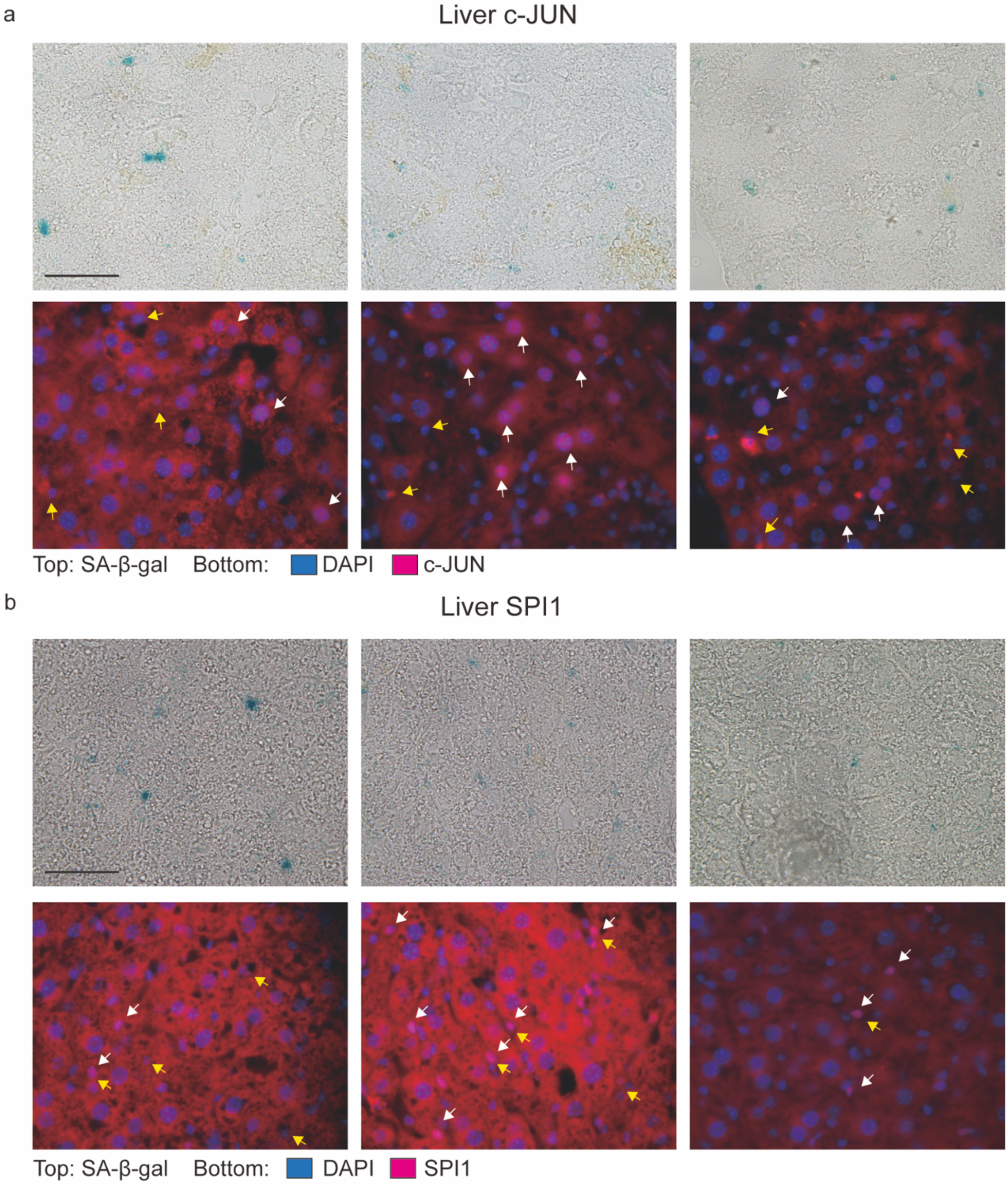

Supplementary Figure 12. The detection of cellular senescence in aged liver.

(a, b) Activation of c-JUN (a) or SPI1 (b) in senescence cells was examined by co-staining for SA-β-Gal activity (top) and TFs by immunofluorescence (bottom) in aged liver. c-JUN^{+} nuclei was rarely co-localized with active SA-β-gal, while some of the SPI1^{+} nuclei showed co-localization with SA-β-gal in the interstitial space of the hepatic cells.

The regions of liver were randomly photographed. c-JUN^{+} or SPI1^{+} nuclei are labeled with white arrows and active SA-β-gal nuclei are labeled with yellow arrows. Scale bar = 50 μm.
