# Supplementary material for: AP‐1 is a regulatory transcription factor of inflammaging in the murine kidney and liver
Source: Aging Cell. 2023 May 8;22(7):e13858. doi: 10.1111/acel.13858 (PMC10352569; doi:10.1111/acel.13858)
Supplement: Supplementary file 2 — Table S1 [file ACEL-22-e13858-s001.pdf]

Supplementary Table 1

Gene	Forward Primer (5′ to 3′)	Reverse Primer (5′ to 3′)
Spi1	ATTCGCCTGTACCAGTTCCTGC	TGGACGAGAACTGGAAGGTACC
Spib	CAAGCGCATGACGTATCAGAA	GCTGTCAAACTGGTAGGTGAG
Junb	CTGAAACCCACCTTGGCGC	CCGAAAAGTAGCTGCCTGCC
Il7r	GCGGACGATCACTCCTTCTG	AGCCCCACATATTTGAAATTCCA
Jchain	GAACTTTGTATACCATTTGTCAGACG	CTGGGTGGCAGTAACAACCT
H2-ob	AGGCGGACTGTTACTTCACC	ATCCAGGCGTTTGTTCCACTG
Cd37	GTCCTTTGTGGGTTTGTCCTT	GAGACAGCGCAGCTCCTTTAG
Tlr2	TCTGGGCAGTCTTGAACATTT	AGAGTCAGGTGATGGATGTCG
Cxcr4	AGGAAACTGCTGGCTGAAAAGG	GGAATTGAAACACCACCATCCA
Ccl5	TGCCCACGTCAAGGAGTATTTC	AACCCACTTCTTCTCTGGGTTG
Cdkn2a	GCCCAACGCCCCGAACTCTTTC	GCGACGTTCCCAGCGGTACACA
Batf	CCAGAAGAGCCGACAGAGAC	GAGCTGCGTTCTGTTTCTCC
Ces1d	GGAGAGTCAGCAGGAGGTTTC	GAGGGACACACCACTCTCAG
Abcd3	GGCCTGCACGGTAAGAAAAGT	CCGCAATAAGTAACAAGTAGCCT
Acsm2	ACTAATACCCATACAGTGGGGC	CTGAAGATCTCTTGCCAGCCTTC
Ehhadh	TGGCTCTAACCGTATGGTCC	CTATGATCCGCCTCTGCAA
Me1	AGTATCCATGACAAAGGGCAC	ATCCCATTACAGCCAAGGTC
Acot12	CCACCACCTTGGAGAAGATAAA	GTGAGGTCAGACAAGAGATGATAAG
Ugt2b38	TGCGCCACAAAAGGGCTAA	ACACAAGAGAGTAGGAAGCCG
Fmo2	ACTCAGAGCAACGGAAAGGA	CCTGGGAATGACTTGAGTGG
Nr1h4	GCAGGGAGAAAACGGAAC	TCTGTACATGACTGGTTGCC
Acsm5	CAGGTTCCCAATAACCACCCT	CCGTCAAACAGTGCCTCAG
Crot	GAACGGACATTTCAGTACCAGG	CTTCATTTGCGAATGGTTTCACT
Coq10b	GAGGACTATCAGCATTTCGTTCC	AGTGCGGTTTTACCAAGGTTAC
c-Jun	GCCTACGGCTACAGTAACCC	GAGAAGGTCCGAGTTCTTGG
c-fos	GGGGACAGCCTTTCCTA	CTGTCACCGTGGGGATAAAG
Jund	GAAACGCCCTTCTATGGCGA	CAGCGCGTCTTTCTTCAGC
Il-6	GTTCTCTGGGAAATCGTGGA	GGTACTCCAGAAGACCAGAGGA
Gapdh	CTTTGTCAAGCTCATTTCCTGG	TCTTGCTCAGTGTCCTTGC
